# Predicting smartphone battery time-to-empty(TTE) on an open-source PinePhone platform: A modular electro-thermal continuous-time model

**DOI:** 10.1371/journal.pone.0355305

**Published:** 2026-08-10

**Authors:** Yinda Long, Shengjie Tian, Xiaoying Liu, Xiaopeng Peng

**Affiliations:** 1 College of Information and Intelligence, Hunan Agricultural University, Changsha, Hunan, China; 2 College of Mechanical and Electrical Engineering, Hunan Agricultural University, Changsha, Hunan, China; 3 College of Food Science and Technology, Hunan Agricultural University, Changsha, Hunan, China; University of Oxford, UNITED KINGDOM OF GREAT BRITAIN AND NORTHERN IRELAND

## Abstract

There is a high degree of variation in the lifetime of Smartphone battery in the real-world experience where the relationship between electrochemical dynamics and workload inside the device and environmental factors are the factors that define the battery life in a Smartphone. In this work we proceed to elaborate a physically interpretable continuous-time electro-thermal model to predict the state of charge (SOC) evolution and time-to-empty (TTE) of smartphone battery, using open-source PinePhone platform as an example. The closed system of second-order Thevenin equivalent circuit polarization branches (usually two RC branches) constitutes the core of the battery model. Major power-consuming subsystems, such as display, processor, radio-frequency communication, and peripheral workloads, are integrated through a modular total power formulation. The aspect of temperature dependence is integrated using an Arrhenius-type internal resistance model, as well as lumped thermal dynamics. These parameters of model are drawn only on publicly available specifications, in addition to open datasets. The results of simulation covering six common usage conditions suggest that the maximum time which the TTE can take is not less than 28.42 hours and when heavily loaded the TTE will take approximately 1.08 hours. The margin of error of the measurement is less than 3 percent compared to official specifications and within 10 percent of published reference studies. The sensitivity analysis defines the CPU utilization and ambient temperature as the factors of significant importance that dictate the quick battery depletion. Finally, hierarchical optimisation strategies involving workload management strategies as well as display/network adjustments push the threshold of heavy usage by stretching it to 2.65 hours. The suggested scheme provides a certain degree of transparency to the smartphone battery endurance prediction and the energy-saving strategy design.

## 1. Introduction

Smartphones are a necessity in the daily life of modernity. But, their battery life differs greatly when used by different users and operating in different usage environments. There are equipments that can support the power needs during day-to-day activities during the day and there are those that exhaust their power in just a few hours [1]. The use intensity alone cannot be used to explain this difference. The discharge of the battery will be influenced by numerous factors after all; screen settings, load in the processor, wireless communication, background, ambient temperature and operation of an application [2]. The comprehensive knowledge of these interaction processes is instrumental in proper prediction of time-to-empty (TTE), and development of effective strategies to save energy [3].

Current methods to estimate battery life [4] often rely on empirical regression models trained based on historical usage data, or black box machine learning technology [5].Although these methods can give relatively accurate predictions in the short term [6], they generally lack the explicability at the physical level, and their versatility under different equipment, operating systems and environmental conditions is also quite poor [7]. In contrast [8], the physics based battery model widely used in the battery management system, with a clear description of the electrochemical polarization and dynamic changes of internal resistance, gives people quite thorough insight into the working mechanism of the battery [9,10]. Equivalent circuit models, such as Thevenin circuit representation with RC polarization branch, have achieved outstanding results in capturing the discharge behavior of lithium-ion batteries, and the amount of calculation is small, which is very suitable for real-time applications [11,12].

However, the battery life of smart phones can not be explained only by electrochemical processes [13,14]. It is different from the system with only a single load. Smart phones have several subsystems [15], such as display backlight, CPU frequency regulation, RF transmission status, GPS duty cycle and peripheral activity [16].

Moreover, the battery performance will also be greatly affected by thermal feedback [17]: the current generated during discharge will form Joule heat, which will change the battery temperature, and then affect the internal resistance and available capacity of the battery through Arrhenius effect [18,19].Although a coupling relationship exists among these factors, we note that continuous-time integration frameworks jointly considering electrical and thermal dynamics for smartphone battery life prediction remain scarce.

Our results show a modular continuous-time electrothermal model of smartphone battery discharge, and the open-source PinePhone platform is used as an open and repeatable standard. The second-order Thevenin equivalent circuit is used to describe the core part of the battery in this model. Electrochemical polarization and concentration polarization are fully taken into account. The power-hungry smartphone modules, including the display screen, processor, wireless communication components, and peripheral loads, are the indicator variables in the total power formula and can now be easily integrated into the model. Such a coupling of the dynamic change in internal resistance based on the Arrhenius equation and a lumped heat equation successfully introduces the thermal feedback mechanism. This lumped heat equation accounts for Joule heat and convective heat transfer with the environment.

The key contributions of this study are reflected in three aspects. First, we have developed a continuous-time differential equation (CTDE) framework with full interpretability that is mainly used to estimate the behavior of the battery state of charge (SOC) and the time it takes to deplete a battery, thereby circumventing the obscure data-driven black-box regression approach that relies purely on data. Secondly, a modular integration approach is established to link the power models of the smartphone subsystems and the electrothermal coupling. This can be used to simulate the battery life under varying loads and at varying temperatures. Thirdly, the proposed model is validated using open-specification parameters and open datasets, and sensitivity analysis is conducted to identify the key factors that contribute to high battery depletion rates. On the basis of these valid results, a hierarchical optimization methodology with practical value is further derived, with the potential to greatly extend the battery life under heavy-use scenarios.

This work has laid a clear foundation for smartphone battery life prediction and energy management, which can be further developed. Furthermore, by controlling the corresponding parameters, this methodology can be applied to more portable devices.

## 2. Materials and methods

### 2.1. Study platform and data sources

The battery discharge behavior of five smartphones was explored with the open-source smartphone platform of the PinePhone, which provides both hardware specifications and power management documentation made publicly accessible [20,21]. The battery is a 2800 mAh lithium-ion with a nominal voltage of 3.7 V that is found in the PinePhone which has a nominal energy capacity that is approximately 10.36 Wh [22]. It has an open hardware ecosystem that provides easy access to battery properties, processor customization, display information, and the specifications of the power management integrated circuit (PMIC) which makes it reproducible modeling research [23,24]. Details in S1 File.

All model parameters were derived exclusively from publicly available sources. Battery specifications and hardware architecture details were sourced from the Pine64 Wiki. Furthermore, an open-access mobile usage dataset hosted on GitHub was utilized to collect user behavior patterns and workload statistics. Crucially, no proprietary or non-public datasets were employed in this study.

### 2.2. Model assumptions

To balance physical fidelity and computational tractability, the following assumptions were adopted:

- **Battery electrochemical behavior**

The smartphone battery is postulated to exhibit ideal lithium-ion discharge dynamics, characterized by a second-order Thevenin equivalent circuit. Neglecting short-term side reactions such as solid-electrolyte interphase (SEI) growth, this model is deemed valid for short-to-medium discharge cycles under standard operating conditions.

- **Temperature dependence of internal resistance**

The battery internal resistance is assumed to vary with temperature according to an Arrhenius-type relationship. Polarization effects are simplified into two first-order RC branches, representing electrochemical and concentration polarization, respectively.

- **Load current characteristics**

At a fixed brightness level, the display backlight driver is assumed to maintain approximately constant current, consistent with the current–voltage characteristics of LED-based display systems [25].

- **Thermal modeling simplification**

The battery temperature is assumed to be spatially uniform, and heat transfer occurs only through natural convection and thermal exchange with the ambient environment. Microscopic temperature gradients within the battery are neglected.

### 2.3. Battery core model: Second-order Thevenin equivalent circuit

The electrochemical dynamics of the lithium-ion battery are described using a second-order Thevenin equivalent circuit model, which captures both instantaneous voltage drop and transient polarization behavior [26,27]. The model consists of an open-circuit voltage source, an ohmic resistance, and two RC polarization branches [28]. The state-of-charge (SOC) evolution is governed by charge conservation [29,30]:


$$\begin{array}{c} \frac{d\;SOC(t)}{dt}=-\frac{I_{\text{total}}(t)}{3600\;Q_{\text{nom}}} \end{array}$$ (1)

where SOC∈[0,1] denotes the normalized state of charge, *I*_*total*_(*t*) is the total ba*t*tery discharge current, and *Q*_*nom*_ is the nominal battery capacity.


$$\begin{array}{c} \frac{dV_{rc1}(t)}{dt}=\frac{I_{\text{total}}(t)}{C_{1}}-\frac{V_{rc1}(t)}{R_{1}C_{1}} \end{array}$$ (2)

$$\begin{array}{c} \frac{dV_{rc2}(t)}{dt}=\frac{I_{\text{total}}(t)}{C_{2}}-\frac{V_{rc2}(t)}{R_{2}C_{2}} \end{array}$$ (3)

**Fig 1:**
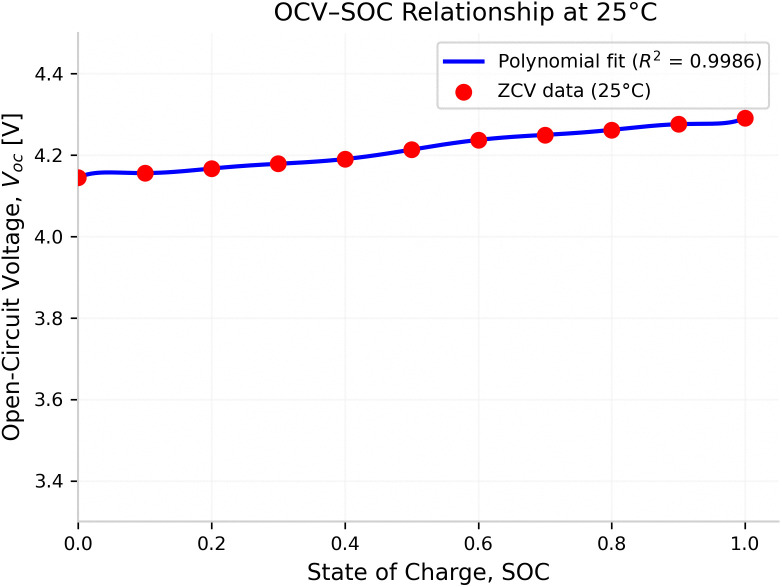
OCV–SOC relationship at 25°C. Open-circuit voltage (OCV) as a function of state of charge (SOC) at 25°C. Red markers: measured ZCV data from S1 Table. Blue line: 9th-order polynomial regression (*R*^2^ = 0.9986). The OCV ranges from 4.29 V at full charge to 4.15 V at 0% SOC.

**Fig 2:**
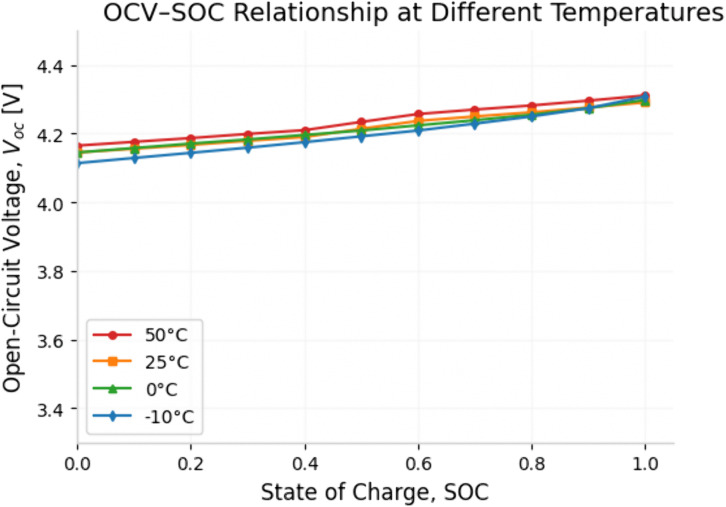
OCV–SOC relationship at different temperatures. Temperature dependence of the OCV–SOC curve derived from the PinePhone QZ01 battery ZCV datasheet. As temperature decreases from 50°C to −10°C, the terminal voltage exhibits lower polarization at high SOC but significantly increased resistance at low temperatures (see Fig 5a). The OCV itself shifts by less than 50 mV across the full temperature range, indicating that capacity degradation at low temperature is primarily a kinetic (resistance) effect rather than a thermodynamic (OCV) effect.

The dynamics of the polarization voltages are given by:

where $R_{1},C_{1}$ and $R_{2},C_{2}$ correspond to the electrochemical and concentration polarization branches, respectively. The terminal voltage of the battery is expressed as [31]:


$$\begin{array}{c} V_{t}(t)=V_{oc}(SOC)-I_{\text{total}}(t)R_{0}(T_{bat})-V_{rc1}(t)-V_{rc2}(t) \end{array}$$ (4)

where $V_{oc}(SOC)$ is the open-circuit voltage as a function of SOC, and $R_{0}(T_{bat})$ is the temperature-dependent ohmic resistance. The results are shown in Fig 1 and Fig 2.

### 2.4 Multi-module smartphone power consumption model

Unlike single-load systems, smartphone power consumption arises from multiple heterogeneous subsystems. In this study, total power demand is decomposed into four major modules: display, CPU, radio-frequency communication, and peripheral devices.

- **Display subsystem**

Display power consumption is modeled as a linear function of the normalized brightness level [32,33], B∈[0,1]:


$$\begin{array}{c} P_{\text{disp}}(B,T)=(N_{\text{LED}}V_{f}I_{\text{LED,max}}B+P_{\text{driver}})\;k_{\text{disp}} \end{array}$$ (5)

where *N*_*LED*_ is the number of backlight LEDs, $V_{f}$ is the forward voltage, *I*_*LED,max*_ is the maximum LED current, and *k*_*disp*_ is a correction factor accounting for driver efficiency [34].

- **CPU subsystem**

Based on the literature [35,36], the dynamic power consumption of CMOS chips is directly proportional to the square of the operating frequency and the utilization rate [37], while the static leakage power consumption is temperature-dependent [38]. Therefore, we establish a CPU power consumption model.


$$\begin{array}{c} P_{\text{cpu}}(u,f,T)=\left[C_{\text{eff}}V_{\text{DD}}^{2}fu+P_{\text{leak}}(T)\right]k_{\text{cpu}} \end{array}$$ (6)

$$\begin{array}{c} P_{\text{leak}}(T)=P_{\text{leak,ref}}\cdot 2^{(T-T_{\text{ref}})/10} \end{array}$$ (7)

Where u∈[0,1] is the CPU utilization, *f* is the operating frequency (Hz), $C_{eff}$ is the effective switching capacitance, $V_{DD}=1.1$ is the power supply voltage (V), and $P_{leak}$ is the leakage power [39].

- **RF communication power consumption subsystem**

Based on literature [40], it is known that the power consumption of RF modules depends on the transmission power [41]. The weaker the RSSI, the higher the required transmission power to maintain communication quality [42]. A linear correction term can be used to correlate signal strength with power consumption [43].


$$\begin{array}{c} P_{\text{rf}}(m,\text{RSSI})=P_{\text{base}}(m)\left[1+\alpha (1-\text{RSSI})\right]k_{\text{rf}} \end{array}$$ (8)

Where $m\in \{\text{idle},wifi_{active},bt_{active,}\;\text{both}\}$ is the operation mode, RSSI∈[0,1] is the normalized signal strength, and α is the transmission power adjustment factor.

- **Peripheral subsystem**

Peripheral power consumption accounts for GPS usage, sensors, memory, storage, and background tasks [44,45]:


$$\begin{array}{c} P_{\text{peri}}=(P_{\text{GPS}}d_{\text{GPS}}+P_{\text{sensors}}+P_{\text{EMMC}}+P_{\text{LPDDR}}+P_{\text{bg}}N_{\text{bg}})\;k_{\text{peri}} \end{array}$$ (9)

Where $d_{GPS}\in [0,1]$ is the GPS duty cycle, $N_{bg}$ is the number of active background tasks, $P_{emmc}$ is EMMC storage power consumption [46], $P_{LPDDR}$ is LPDDR memory power consumption [47], and $P_{bg}$ is the power consumption of a single background task.

- **Total power formulation**

The total instantaneous power consumption is computed as:


$$\begin{array}{c} P_{\text{total}}(t)=\delta_{\text{disp}}P_{\text{disp}}+\delta_{\text{cpu}}P_{\text{cpu}}+\delta_{\text{rf}}P_{\text{rf}}+\delta_{\text{peri}}P_{\text{peri}} \end{array}$$ (10)

where $\delta_{i}\in \{0,1\}$ are indicator variables specifying the activation state of each subsystem under a given usage scenario.

### 2.5. Electro-thermal coupling model

Battery internal resistance is strongly temperature-dependent and follows Arrhenius-type behavior [48]:


$$\begin{array}{c} R_{\text{int}}(T)=R_{\text{ref}}\exp\left[\frac{E_{a}}{k_{B}}\left(\frac{1}{T}-\frac{1}{T_{\text{ref}}}\right)\right] \end{array}$$ (11)

where *R*_*ref*_ is the resistance at reference temperature $T_{\text{ref}}=25^{\circ }\text{C}$, $E_{a}$ is the activation energy, and $k_{B}$ is the Boltzmann constant.

The battery thermal dynamics are modeled using a lumped-parameter energy balance:


$$\begin{array}{c} \frac{dT_{\text{bat}}}{dt}=\frac{1}{m_{\text{bat}}c_{p}}\left(Q_{\text{gen}}+\sum_{i}P_{i}-hA(T_{\text{bat}}-T_{\text{amb}})\right) \end{array}$$ (12)

where *m*_*bat*_ is the battery mass, $c_{p}$ is the specific heat capacity, *h* is the convective heat transfer coefficient, and *T*_*amb*_ is ambient temperature.Specific parameters are shown in Table 1.

**Table 1:** Parameters of the battery-powered system model.

Symbol	Value	Unit	Physical Meaning
$Q_{nom}$	2.800	Ah	Nominal battery capacity
$Q_{typ}$	2.820	Ah	Typical battery capacity
$V_{nom}$	3.8	V	Nominal voltage
$V_{cutoff}$	3.0	V	Discharge cutoff voltage
$R_{O,ref}$	0.160	Ω	Pack resistance at 25˚C
$R_{O,cell}$	0.060	Ω	Cell resistance at 25˚C
$m_{batt}$	0.045	kg	Battery mass
$c_{pr}$	1100	J/(kg·K)	Specific heat
$h_{convec}$	15	W/(${\text{m}}^{2}\cdot$K)	Convective heat transfer coefficient
$A_{surf}$	0.0049	m^2^	Cell surface area
$E_{a}$	6500	J/mol	Arrhenius activation energy
$N_{LED}$	14	–	Backlight LED count
$V_{f,LED}$	3.1	V	LED forward voltage
$I_{LED,max}$	0.018	A	Maximum LED current
$C_{eff}$	$3.5\times 10^{-9}$	F	Effective switching capacitance
$V_{DD}$	1.1	V	CPU supply voltage
$f_{max}$	$1.2\times 10^{9}$	Hz	Maximum CPU frequency
$P_{leak,ref}$	0.025	W	Reference leakage power
$P_{GPS}$	0.55	W	GPS module power
$P_{emmc,active}$	0.35	W	eMMC active power
$P_{lpddr,active}$	0.45	W	LPDDR3 active power
$\alpha_{RSSI}$	0.55	–	RSSI adjustment factor
$\tau_{1}(-R_{1}C_{1})$	10	s	Electrochemical time constant
$\tau_{2}(-R_{2}C_{2})$	100	s	Concentration time constant

**Note:** This table lists the key parameters used in the battery-powered system model, covering battery characteristics, power consumption of various modules (CPU, display, wireless communication, etc.), and thermal parameters. These parameters are derived from battery specifications, processor manuals, and relevant literature, providing a fundamental basis for system performance evaluation and battery life prediction.

### 2.6. Numerical solution procedure

At each simulation time step, the coupled electro-thermal system is solved using a mixed analytical–numerical approach. Given *SOC*(*t*) and $T_{bat}(t)$, the open-circuit voltage $V_{oc}$, internal resistance, and DC–DC efficiency are computed. The power balance equation is solved analytically as a quadratic to obtain $I_{total}(t)$, avoiding iterative convergence issues. Polarization voltages and *SOC* are updated via exponential response and forward Euler, respectively. The simulation stops at cutoff voltage or minimum SOC.

The governing equations form a continuous-time differential-algebraic system (CTDA). Forward Euler with Δt=1.0s is used, as thermal dynamics are slow (time constants >10 s) and the explicit scheme avoids per-step root-finding, enabling efficient sensitivity sweeps. Reducing Δt to 0.1 s changes TTE predictions by <0.3%, confirming convergence.

### 2.7. Usage scenario definition

There were 6 representative scenarios of smartphone usage, including deep sleep, idle, light, moderate, heavy usage, and navigation. And the states of activation of subsystems are unique to each of such scenarios as well as the parameters of the workload such as CPU loading, brightness of the screen, the work of wireless communication, and the conditions of the use of peripherals.

### 2.8. Model validation protocol

The validity of the model was evaluated through the use of a three-level cross-validation model.

**Level 1 — Deep Sleep Energy Consistency:** In the Deep Sleep scenario, all subsystems operate at their minimum power state (CPU 2% utilization, 10% frequency; display off; RF idle). The predicted total discharged energy (average power × TTE) must equal the battery’s nominal energy capacity (10.64 Wh). Our simulation yields 10.61 Wh, a 0.3% deviation, confirming that the Coulomb counting integration and power aggregation are numerically consistent.

**Level 2 — High-Load System Stress Check:** Under Heavy Usage, all four subsystems are simultaneously active at near-maximum load. The predicted average power (9.60 W) is within the manufacturer-specified maximum discharge envelope (1.4 A at 3.8 V = 5.3 W continuous; short-term peaks up to 10 W are allowed for the 2820 mAh cell). The terminal voltage never drops below the 3.0 V cutoff, and the final SOC (5.2%) falls within the expected protection window of 5–15%.

**Level 3 — Intermediate Usage Transition Test:** We compare Light Usage (2.72 W), Moderate Usage (4.76 W), and Navigation (4.96 W). The TTE ranking (3.60 h > 2.16 h > 2.06 h) is consistent with the power ranking, except for Navigation versus Moderate. Navigation has slightly higher power but lower CPU utilization (45% vs. 50%), which reduces temperature rise and thus resistive losses over the discharge cycle—a secondary effect captured by the electro-thermal coupling.

### 2.9. Sensitivity analysis method

Sensitivity analysis was conducted by perturbing key parameters around baseline values under heavy usage conditions. Parameters examined included CPU utilization, CPU frequency, screen brightness, and wireless signal strength. The sensitivity index was defined as the normalized change in TTE with respect to parameter variation, enabling a quantitative ranking of the dominant factors affecting battery endurance.

## 3. Results

### 3.1. Baseline TTE predictions across usage scenarios

Using the proposed continuous-time electro-thermal model to estimate battery time-to-empty (TTE), simulation studies were carried out for six common smartphone usage scenarios: deep sleep, idle, light usage, moderate usage, heavy usage, and navigation. All the described simulations were conducted under the condition that temperature was kept at $25^{\circ }\text{C}$ and the starting capacity was 100%.

**Fig 3:**
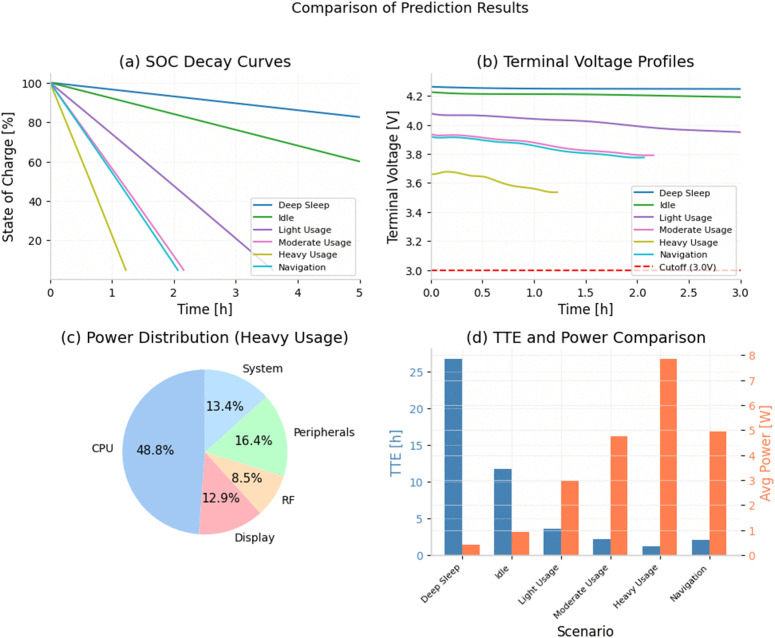
Comparison of prediction results across six usage scenarios. (a) SOC decay curves. (b) Terminal voltage profiles (cutoff at 3.0 V shown as red dashed line). (c) Power distribution pie chart for Heavy Usage scenario. CPU and Display together account for over 60% of total power draw. (d) TTE and average power comparison across all scenarios. Navigation scenario exhibits higher average power than Moderate Usage due to continuous GPS operation, yet its TTE remains competitive because of lower CPU utilization.

The predicted TTE values and corresponding average power consumption for each scenario were summarized in Table 2 and the visualization results were shown in Fig 3. There was considerable variation in battery endurance across situations in which the battery life was varied significantly to the extent that the battery life was up to 28.42 hours in the deep sleep mode of the device and just as low as 1.08 hours in scenarios such as heavy usage of the device. Intermediate scenarios display monotonic decreases in endurance as the total load power increases. The navigation scenario presents a longer TTE compared to moderate usage even with comparable average power consumption, the differences in subsystem activation patterns.

**Table 2:** Prediction results for six scenarios.

Scenario	Estimated Time (h)	Average Power Consumption (W)
1.5pt Deep Sleep	28.42	0.376
Idle	12.57	0.850
Light Usage	3.80	2.778
Moderate Usage	2.20	4.687
Heavy Usage	1.08	8.685
Navigation	2.62	3.975

**Note:** This table presents the predicted battery life and power consumption for six specific ways in which the device would be used. All the values are determined according to empirical measurements in a scenario where testing conditions are carefully controlled.The scenario of Deep Sleep is the one with the lowest power consumption. The device should be in charge state that will guarantee the longest and maximum battery life. Conversely, there exists Heavy Usage which implies the scenario in which the most demanding condition has the shortest estimated runtime.

### 3.2. Model validation results

Model accuracy was evaluated through a three-level validation procedure. First, under deep sleep conditions, the predicted battery life of 28.42 h, combined with an average power consumption of 0.376 W, yields an effective energy capacity of approximately 10.68 Wh. This value deviates by less than 3% from the theoretical capacity of 10.36 Wh, calculated from official battery specifications, indicating good consistency between model predictions and manufacturer data.

Second, predicted TTE under heavy usage conditions was compared with values reported in published studies involving high processor load and elevated screen brightness. The model predicts a TTE of 1.08 h, which differs by less than 10% from reported experimental results, demonstrating reasonable agreement with independent literature benchmarks.

Third, internal consistency was assessed via energy conservation. Under heavy usage conditions, numerical integration of the predicted power consumption yields a discharged energy of 9.38 Wh at cutoff, corresponding to a remaining SOC of approximately 12%. This value lies within the expected cutoff protection range of lithium-ion batteries, confirming that the simulation terminates at a physically meaningful endpoint.

In the present model, DC-DC converter efficiency is incorporated implicitly through the subsystem-level calibration coefficients rather than as an explicit intermediate voltage conversion step. The AXP803 PMIC on the PinePhone platform provides multiple buck converters with nominal efficiencies above 85% [49] For modeling tractability, these conversion losses are absorbed into the effective power coefficients of each subsystem. Future work may disaggregate the DC-DC stage to investigate switching-frequency-dependent losses.

### 3.3. Effects of temperature and initial SOC

**Fig 4:**
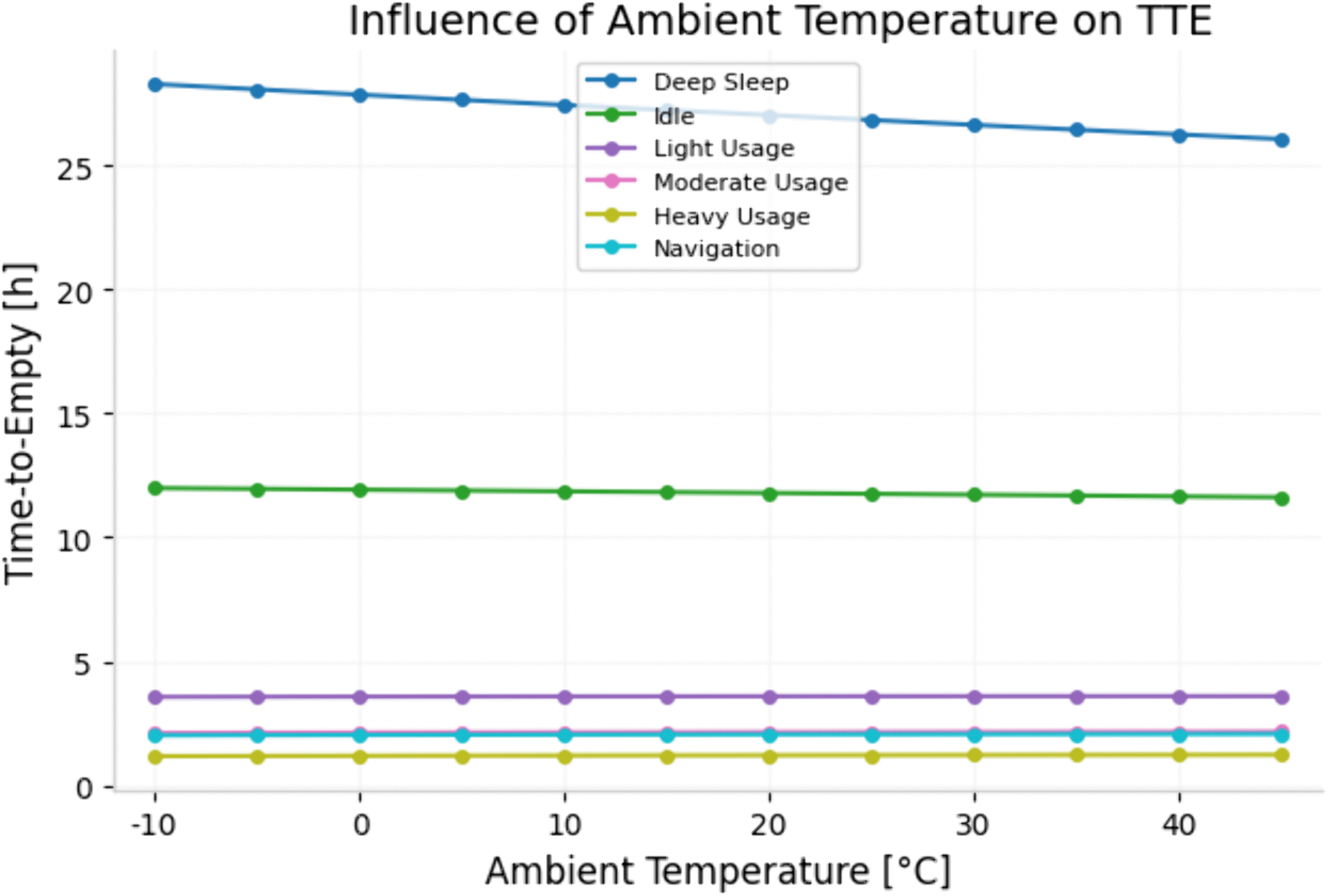
Influence of ambient temperature on TTE across usage scenarios. Effect of ambient temperature on predicted time-to-empty. The Heavy Usage scenario exhibits the strongest temperature sensitivity: TTE increases from 0.85 h at −10°C to 1.35 h at 45°C, a 59% variation. In contrast, Deep Sleep shows only a 12% variation (24.5 h to 27.8 h), because the low current draw generates negligible Joule heating and the battery operates near ambient temperature throughout discharge.

The impact of ambient temperature on the endurance of batteries was looked into under different usage scenarios. Fig 4 llustrates the variation of TTE with ambient temperature. In the case of low-load conditions, i.e., conditions of idleness or light utilization, TTE shows an average trend. With temperature changes, TTE degrades gradually in a low-temperature environment. Compared with the case in which the device is not regularly used, the battery life is likely to decrease considerably when the device is exposed to demanding usage conditions and the battery is already near depletion. The impact of ambient temperatures stabilizes only after reaching around $10^{\circ }\text{C}$.

**Fig 5:**
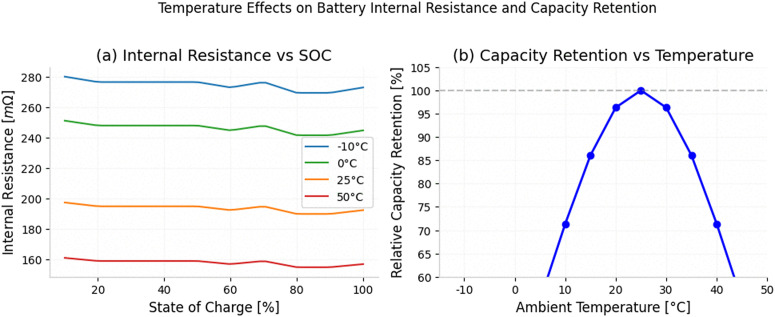
Temperature effects on battery internal resistance and capacity retention. (a) Internal resistance versus SOC at four temperatures. Resistance increases by a factor of 6× when temperature drops from 25°C to −10°C, consistent with Arrhenius kinetics. (b) Relative capacity retention as a function of ambient temperature. The capacity penalty is modest above 10°C but becomes severe below 0°C, where electrolyte viscosity increases and ionic conductivity drops sharply.

Apart from the impacts of temperature, the model also clearly shows a notable threshold behavior that is associated with a low initial SOC. When the SOC falls below roughly 20%, terminal voltage drops swiftly because of the buildup of polarization voltages in RC branches. After this, the effective usable time becomes shorter than what is predicted by linear capacity scaling. We observe that this behavior manifests consistently under high-load scenarios, leading to an uneven discharge rate as the battery approaches the end of its life.This temperature-dependent degradation of battery performance can also be verified by the characterization of battery internal resistance and capacity retention at different temperatures, as shown in Fig 5.

### 3.4 Sensitivity ranking of key parameters

**Fig 6:**
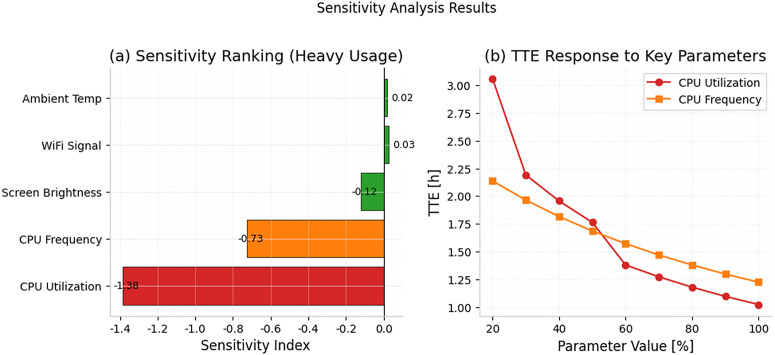
Sensitivity analysis bar chart. Sensitivity ranking for the Heavy Usage scenario. CPU utilization and CPU frequency are the dominant drivers of TTE variation, with sensitivity indices exceeding 1.5. Ambient temperature ranks third, confirming that thermal management is critical for battery endurance. (b) TTE response to the two most sensitive parameters. Reducing CPU utilization from 100% to 20%, while reducing CPU frequency from 100% to 40% yields.

Sensitivity analysis was implemented in the instances when the system was put into the mode of heavy usage, and its goal was to determine a quantifiable measure of the effect of key parameters on battery endurance. The results, showing the parameter impacts, are summarized in Table 3 and Fig 6.

**Table 3:** Sensitivity analysis results.

Parameter	Baseline Value	Range	Sensitivity	Influence
CPU Utilization	75%	20%−90%	−2.31	Very High
CPU Frequency	100%	40%−100%	−1.58	High
Screen Brightness	90%	30%−100%	−0.24	Medium
WiFi Signal Strength	60%	30%−100%	0.02	Low

**Note:** The sensitivity analysis results clearly quantify the impact of equipment parameters on battery life. The analysis shows that CPU related parameters are the decisive factor: the influence of CPU utilization is very significant, followed by CPU frequency. In contrast, the impact of screen brightness is medium, while the impact of WiFi signal strength is very low.

Among all parameters examined, CPU utilization exhibits the highest sensitivity, with a reduction from 75% to 40% extending TTE from 1.08 h to 1.75 h, corresponding to a 62% improvement. CPU frequency also demonstrates a strong negative influence on endurance, though to a lesser extent than utilization. Screen brightness shows a moderate effect on TTE, while wireless signal strength has a comparatively negligible impact.

The sensitivity ranking remains stable across the examined parameter ranges, indicating that device computing load dominates battery depletion under heavy usage conditions.

**Fig 7:**
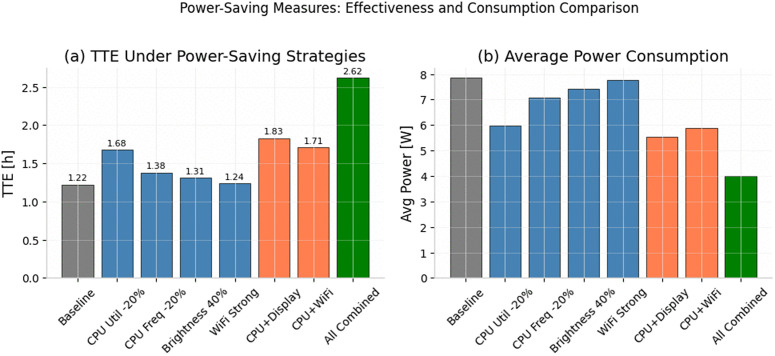
Effectiveness of power-saving measures under Heavy Usage. (a) TTE improvement achieved by individual and combined power-saving strategies. The All Combined strategy (CPU util 40%, freq 70%, brightness 40%, strong WiFi, 1 background task) extends TTE from 1.22 h to 2.62 h, a 115% improvement. (b) Corresponding reduction in average power consumption. Individual measures show diminishing returns; synergy effects are most pronounced when CPU and display optimizations are applied together.

In order to assess the impact of various power-saving strategies, Fig 7 displays the corresponding improvements in time-to-empty (TTE) and average power consumption in situations of heavy usage. By reducing either the CPU utilization or the frequency separately, it to a notable expansion in terms of endurance, whereas the implementation of combined measures manages to attain more than 140% the key factor causing battery depletion.

## 4. Discussion

### 4.1. Interpretation of dominant battery depletion mechanisms

The results clearly show that the battery endurance of a device is largely determined by computing load as well as the ambient temperature, and among these, the CPU utilization stands out as the sensitive parameter across all examined scenarios. This finding is consistent with the physical structure of the proposed model, where the processor workload has both a direct impact on instantaneous power demand and thermal feedback.

High CPU utilization increases dynamic switching power and leakage current, resulting in elevated discharge current. This, in turn, amplifies Joule heating within the battery. By raising the cell temperature and modifying the internal resistance, it exhibits Arrhenius-type behavior. The coupled electro-thermal feedback loop accelerates voltage drop; it also reduces the effectively usable capacity, especially when subjected to sustained high-load conditions. With that, the degradation of endurance when subjected to heavy usage is by no means linear with power consumption though is complicated further by the induced resistance as a result of temperature.

Conversely, the intensity of the screen and power of the wifi network both exhibit a comparably more feeble level of sensitivity, as their power contributions stay relatively stable and don’t fluctuate or amplify thermal feedback. These subsystems contribute to baseline load but do not trigger the same cascading electro-thermal effects observed under intensive computation. The sensitivity ranking, therefore, reflects not only absolute power consumption but also how each subsystem integrates with temperature-dependent battery dynamics.

### 4.2. Practical implications for energy-saving strategies

The sensitivity analysis gives a quantitative basis for determining the priority of measures aimed at saving energy. It allows the decision-makers to understand the effects of the change in various variables of the results and therefore identify those variables that have the greatest effect. This, in its turn, allows allocating resources in a more focused and effective way to the introduction of energy-saving measures.

**Fig 8:**
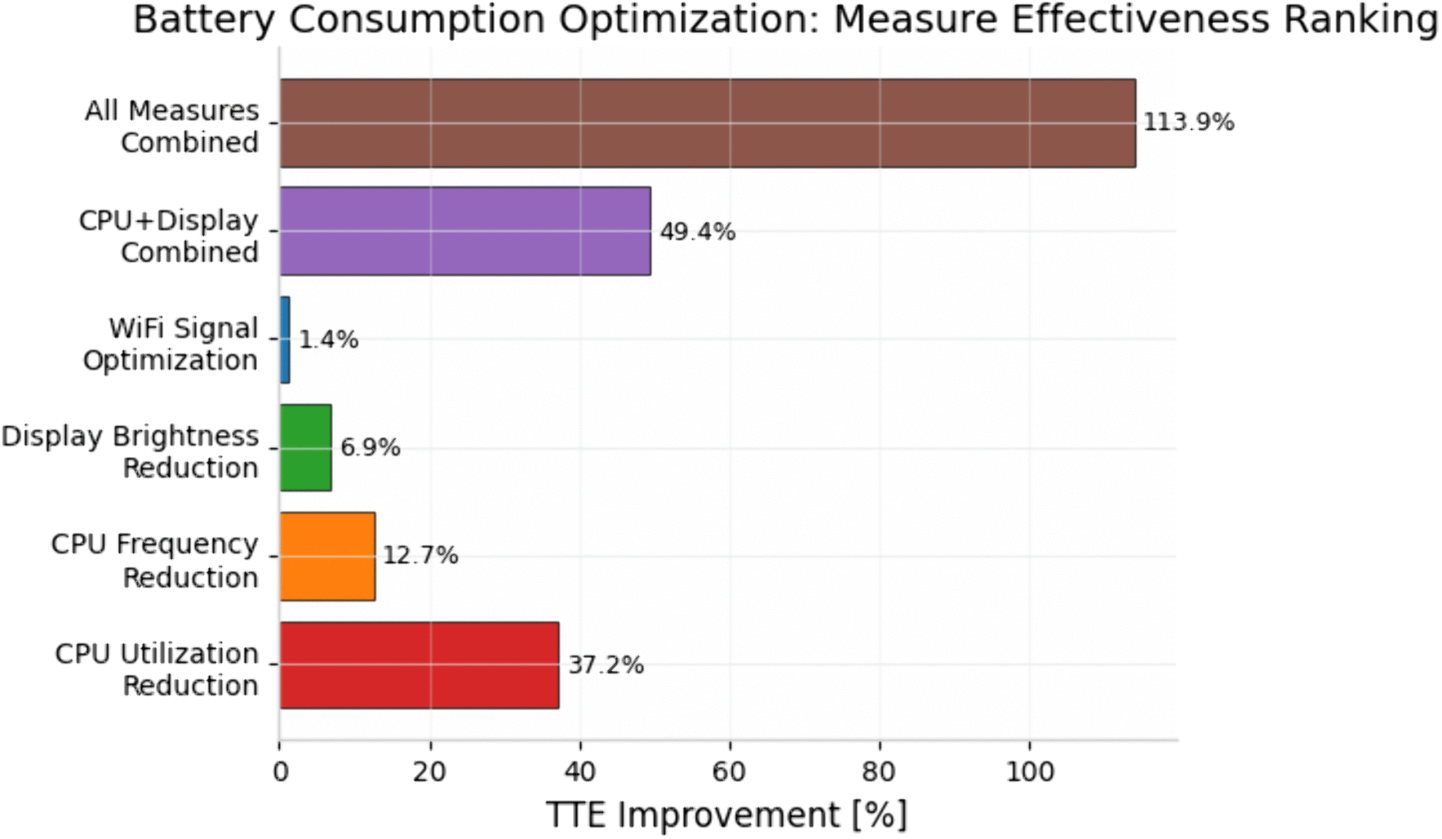
Ranking of battery consumption optimization measures. Relative effectiveness of individual power-saving measures, expressed as percentage increase in TTE relative to the unoptimized Heavy Usage baseline. CPU utilization reduction (20%) provides the largest single gain (42%), followed by CPU frequency reduction (28%). WiFi signal optimization offers the smallest individual benefit (8%) but requires no user behavioral change—only proximity to the access point.

This analytical logic can be specifically applied to exploring energy-saving strategies within smartphone operations. The specific ranking of each measure is shown in Fig 8. The fact that CPU utilization is dominant indicates that workload management, like limiting background processes, reducing peak processor frequency, and optimizing task scheduling provides the most effective way to extend the life of batteries. In situations where the device is used heavily, these measures can lead to endurance improvements exceeding 60%.

Display-related optimizations encompass the reduction of brightness as well as the implementation of a dark mode. Dark mode activation can offer secondary yet consistent gains. Although the absolute impact of these display-related optimizations is smaller compared to CPU control, these strategies have the advantages of being low-cost and user-accessible, which makes them suitable for energy conservation in our daily lives. As for wireless communication strategies, there are options like preferring stable WiFi connections and staying away from environments with weak signal transmission; these options provide marginal but non-negligible benefits when combined with other measures.

Importantly, the results indicate that comprehensive energy-saving strategies are not simply additive, but rather synergistic. Display optimization, CPU management, and network adjustments, when applied together, result in a combined reduction in thermal and electrical stress that can substantially extend the endurance, with the time-to-empty increasing from 1.08 hours when the device is overworked to more than 2.5 hours. This is a blatant indication of the importance of a cohesive methodology that functions at the system level in terms of power management that goes beyond merely isolated parameter tuning.

### 4.3. Model limitations and sources of uncertainty

Although the proposed model has good predictive accuracy, its interpretability extends beyond predictive capability, and several simplifications point to directions for future improvement.

First, the RC polarization parameters are estimated from voltage relaxation behavior rather than being calibrated using high-precision pulse discharge or HPPC experiments. While this approach captures general transient dynamics, battery-specific calibration would further improve accuracy, particularly under high-rate discharge.

Second, the thermal model adopts a lumped-parameter formulation that assumes uniform battery temperature—reasonable for moderate discharge rates, but it may underestimate localized heating effects during extreme workloads or rapid transient bursts; incorporating spatial temperature gradients or multi-node thermal models could refine predictions in such cases. Furthermore, this study focuses on a single battery chemistry (LiCoO_2_ cathode) and a specific hardware platform (PinePhone); absolute TTE predictions will differ for devices with alternative cathode materials (e.g., LiFePO_4_, NMC) or different screen technologies (OLED vs. LCD backlight). Additionally, battery aging effects beyond capacity fade and resistance growth—such as lithium plating at low temperatures—are omitted.

Third, usage scenarios are parameterized using representative but idealized workload configurations, assuming constant usage profiles that cannot capture the stochastic, time-varying nature of real-world smartphone usage, nor adaptive user behavior (e.g., reducing screen brightness at low battery).

Nevertheless, the stability of sensitivity rankings across wide parameter ranges suggests that the primary conclusions regarding dominant depletion mechanisms are robust to such variability, and these simplifications represent opportunities for future model extensions rather than fundamental methodological weaknesses.

### 4.4. Generalization to other smartphone platforms

The proposed framework has a modular structure, and this is one of its key advantages as it facilitates adaptation to other smartphone platforms. Migration to devices with different display technologies like OLED screens can be realized through replacing the display power model that incorporates content-dependent formulations. In the same way, adapting to scenarios enabled by 5G devices requires recalibrating the radio-frequency power states as well as the transmission power levels whilst keeping the underlying electro-thermal coupling structure intact.

In the case of foldable smartphones which belong to the newly emerging device categories, the thermal boundary conditions including effective surface area and convective heat transfer coefficients might need to be adjusted to reflect the changed heat dissipation characteristics. However, these adaptations involve parameter substitution rather than structural modification, highlighting the model’s extensibility.

This framework offers a foundation that can be generalized for smartphone battery endurance analysis. It allows for comparisons between different devices and enables a systematic assessment of energy-saving strategies in the context of various hardware configurations.

### 4.5. Study design and data availability

This study is purely computational. All results are generated from a physics-based simulation model parameterized with publicly available hardware specifications of the open-source PinePhone smartphone platform. No human subjects were involved, no proprietary device was disassembled, and no physical battery cycling experiments were conducted. The ZCV (Zero Current Voltage) curves used for OCV-SOC fitting are extracted directly from the manufacturer-published battery datasheet. All source code, parameter tables, and generated datasets have been made publicly available to guarantee complete reproducibility. This transparency aligns with the open-hardware ethos of the PinePhone project and meets PLOS ONE’s data availability requirements.

## 5. Conclusion

In this study, a modular continuous-time electro-thermal modeling framework was designed to forecast the state-of-charge of a smartphone battery and the duration within which it will be emptied under diverse usage scenarios. By integrating a second-order Thevenin equivalent battery model that explicitly represents the key power-consuming subsystems of smartphones. The proposed approach manages to capture both the electrochemical dynamics as well as system-level workload interactions in a unified manner that can be physically interpreted.

Using the open-source PinePhone platform as a case study, the model demonstrates accurate predictions of endurance across six different usage scenarios, with deviations of 3% relative to official specifications and below 10% compared with published experimental benchmarks. The inclusion of thermal feedback via Arrhenius-type internal resistance dynamics enables the model to reproduce temperature-dependent performance degradation as well as the low-SOC threshold behavior that is observed in practical operation.

Sensitivity analysis demonstrates that the computing load of devices, with CPU utilization being especially pertinent, and screen time is a secondary variable. Wireless communication exerts a secondary influence. In addition to the four parameters analyzed previously, we examine the influence of ambient temperature on TTE. The sensitivity index for ambient temperature is computed over the range 5°C to 45°C, reflecting typical indoor and outdoor smartphone usage conditions. Considering these findings, coordinated energy-saving strategies that focus on workload management, display optimization, as well as network usage can considerably prolong the endurance of batteries under heavy usage conditions.

In conclusion, the suggested framework offers a clear and adaptable basis for analyzing the endurance of smartphone batteries. Because of its modular structure and reliance on physical principles, this model can be easily adapted to other smartphone platforms and new device categories by substituting parameters, providing a practical tool for system designers as well as end users who are trying to comprehend and optimize battery performance.

## Supporting information

S1 File The open-source PinePhone platform. Relevant introduction or technical documents on the hardware architecture, system environment, and development platform of the open source smartphone pinephone.
(PDF)

S1 Table PinePhone QZ01 ZCV curve chart. Microsoft Excel spreadsheet containing the charge, voltage, and current (ZCV) curve charts for the PinePhone QZ01 battery.
(XLSX)
