# Supplementary material for: Predicting smartphone battery time-to-empty(TTE) on an open-source PinePhone platform: A modular electro-thermal continuous-time model
Source: PLoS One. 2026 Aug 10;21(8):e0355305. doi: 10.1371/journal.pone.0355305 (PMC13456260; doi:10.1371/journal.pone.0355305)
Supplement: Supplementary file 1 [file pone.0355305.s001.pdf]

PinePhone

The **PinePhone** is a smartphone created by PINE64. It is capable of running mainline Linux and is supported by many partner projects.

The Braveheart Edition of the PinePhone was the first publicly available version of the phone. It shipped without a fully functional operating system and was geared specifically towards early adopters. The Braveheart Edition's successors were the Community Editions, which featured a branded backcover and box of selected community projects. The Community Editions became available in June 2020. The Beta Edition featuring Manjaro with Plasma Mobile is the latest edition, it became available in March 2021.

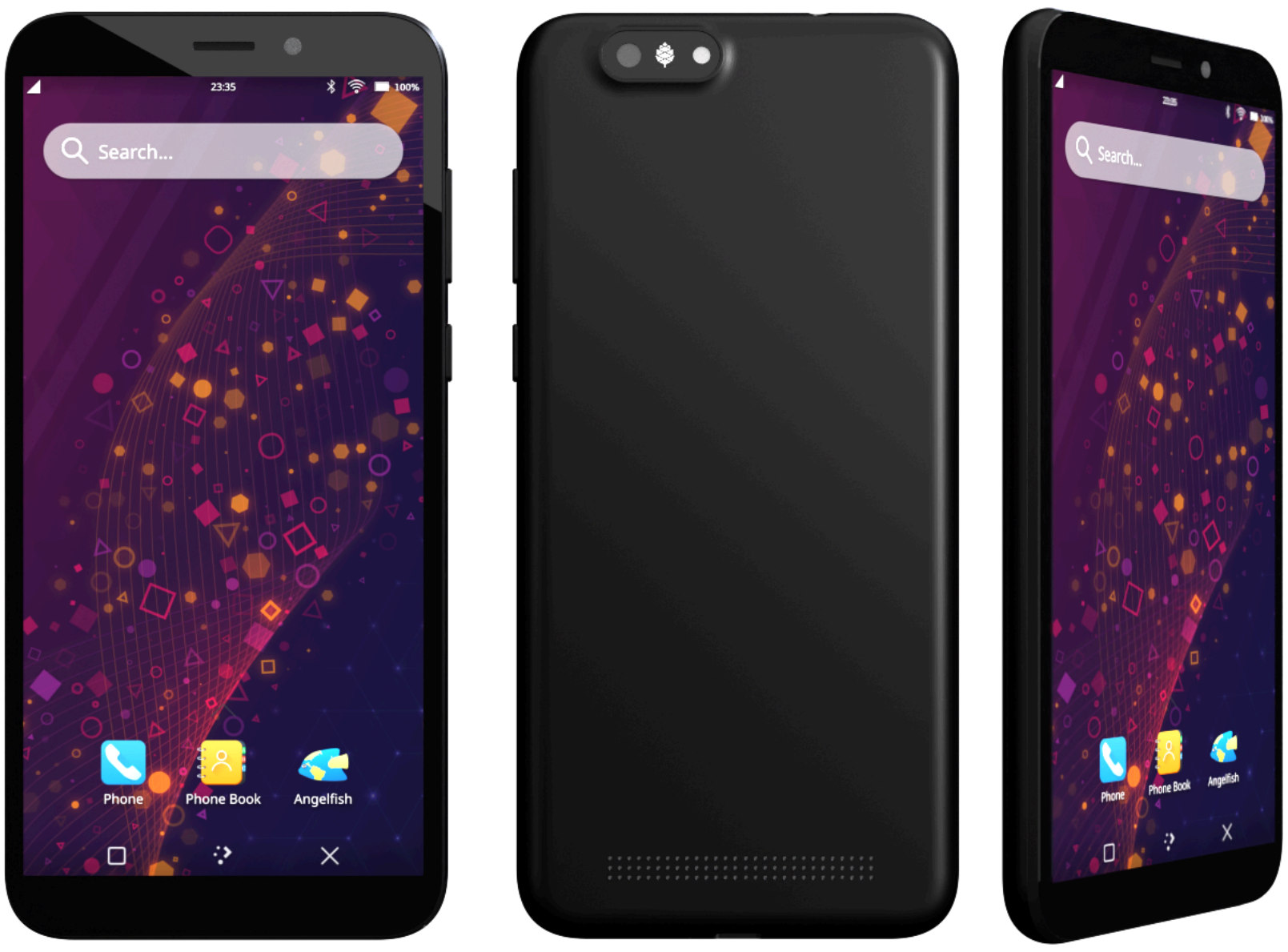

Rendering of the PinePhone Beta Edition

Contents

- Introduction
  - State of the software
  - Help and support
- First time installation
- Operating systems
  - Software releases
  - Installation instructions
  - Troubleshooting
    - The battery is drained
    - The installation is corrupted or incorrect
- Specifications
- Components
- Hardware revisions
- Hardware accessory
  - Add-ons
  - PinePhone Accessories

    - Pogo pins
    - Back cover
    - Serial console
- **Privacy switch configuration**
- **Camera**
- **Battery**
- **Modem**
    - AT commands
    - VoLTE
    - APN settings
    - Carrier support
    - Documents
    - Firmware update
        - Custom firmware
        - Stock firmware
    - Firmware modifications
    - GPS / GNSS
    - Voice mail
- **Safety**
    - General recommendations
    - Charging safety
    - Thermal safety
- **Frequently asked questions**
- **Modifications and repairs**
    - Swapping in a new mainboard revision
        - Replacing the mainboard
        - Flashing the ANX firmware
            - Method 1
            - Method 2
    - Replacing the screen
    - Spare parts not available in the PINE64 store
    - Other hardware issues
- **Press**
- **PinePhone board information, schematics and certifications**
- **Datasheets for components and peripherals**

Resources

External links

Introduction

The PinePhone is not a regular phone and you might not get the latest and greatest hardware and this years' newest innovation. You will get a device with good mainline support with a great community behind it.

State of the software

First things first, the PinePhone is aimed solely at early adopters - more specifically, the units are solely intended to find their way into the hands of users with extensive operating system experience.

Bear in mind that the software for these smartphones is very early, with most of the software being in alpha or beta state. That's especially also the case for scalability of applications, their availability and practicability, any hardware function implementations and the firmware. The software is provided as is. There is no warranty for the software, not even for merchantability or fitness for a particular purpose.

If you have any questions regarding the current state of the software or of specific features working, please don't hesitate to ask in the community chat (see Main Page#Community and Support)!

Help and support

Still have any questions regarding software, shipping, or ordering after reading this wiki? Please don't hesitate to contact the community in the bridged community channels for detailed answers or simply to chat with friendly people in the community! See Main Page#Community and Support.

Please keep in mind that PINE64 is not like a regular company (see the PINE64 philosophy (https://www.pine64.org/philosophy/)) and that support resources are limited - the best way to get support quickly is to ask in the community chat! Please only contact the PINE64 support directly if questions couldn't be solved via the community chat or this wiki.

First time installation

When shipped the battery is isolated from the device using a protective plastic tab, which is required to be removed before using the phone. The battery **will not** charge or boot until it is removed and the battery is connected again.

> **Note:** To remove the sticker after unboxing the phone: Carefully remove the back panel using the notch in the corner of the back cover without overbending it. Then remove the battery. Peel off the clear plastic sticker below it, which isolates the charging contacts and reinsert the battery.

The PinePhone's SIM slot only accepts a micro-SIM, please do not insert a nano-SIM without an adapter and make sure that the nano-SIM does not get released from its adapter. The SIM card has to be placed in the lower slot, while the microSD has to be placed in the upper slot.

> **Note:** Do not insert an empty micro-SIM adapter into the phone and do not release the nano-SIM inside the adapter, as it will get stuck on the contact pins. If the nano-SIM got released inside the adapter inside the phone, carefully reinsert the nano-SIM card without moving the adapter. In that case do not pull on the empty adapter as it will get stuck on the contact pins and damage them!

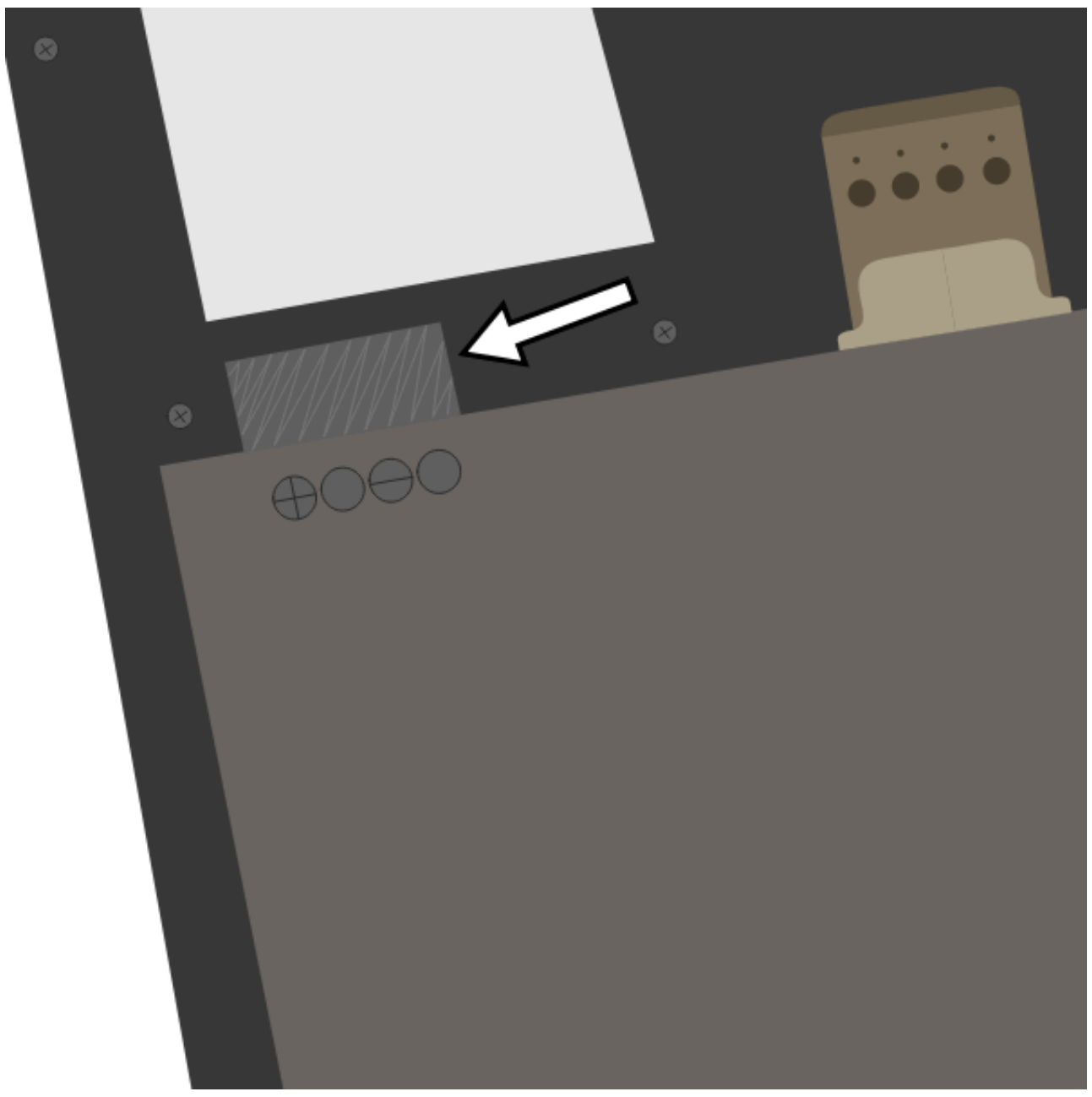

A protection foil isolates the battery for the shipping.

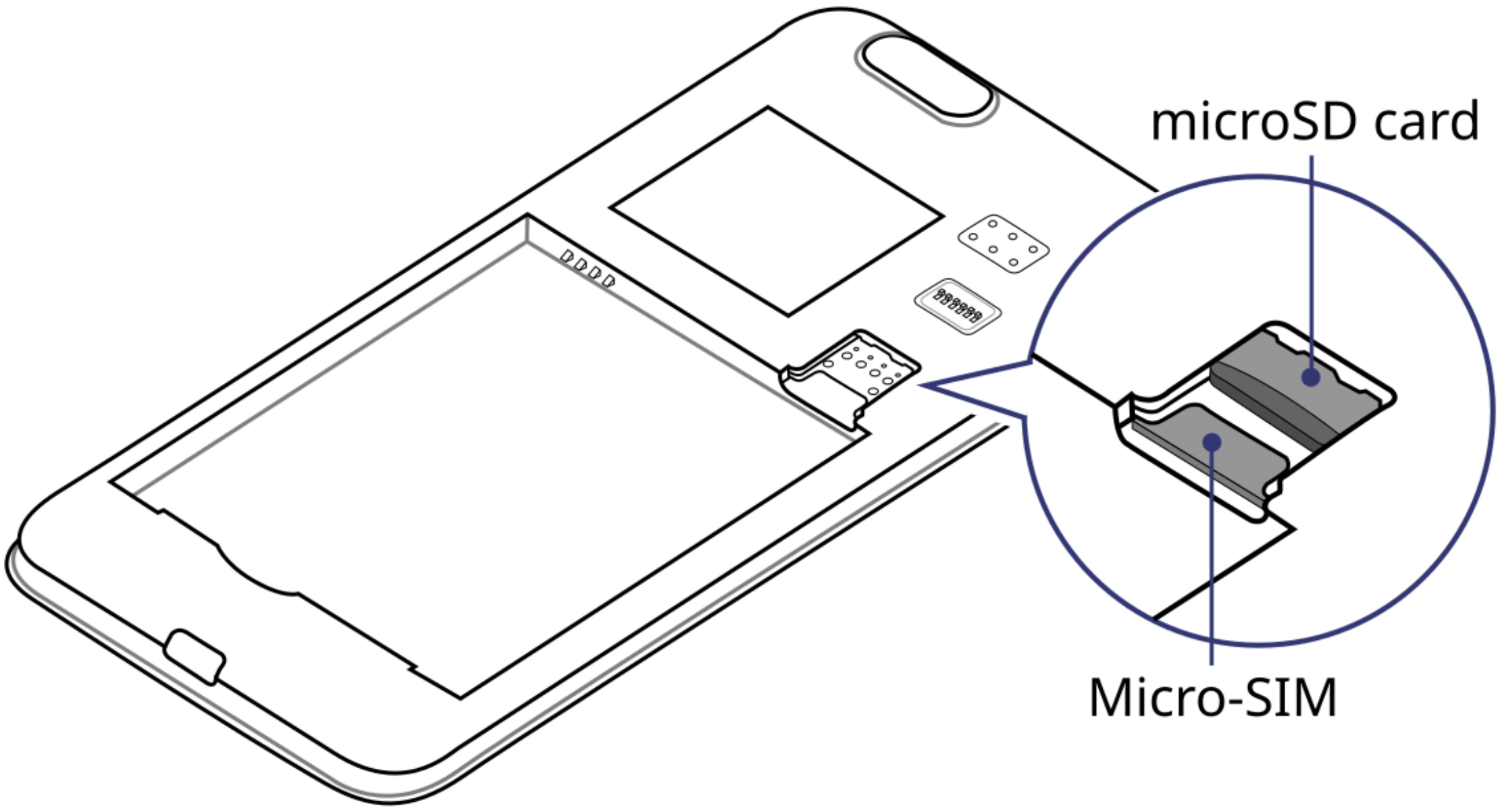

The microSD belongs in the upper slot, the micro-SIM in the lower slot.

An adapter from a nano to a micro-SIM might be included under tape in the camera notch of the phone's packaging. Some nano-SIMs will not fit firmly into that adapter that comes with the PinePhone and if the included adapter is used without a well-fitting nano-SIM, the contact pins might get damaged. In that case it is highly recommended to acquire a better fitting adapter.

Operating systems

The PinePhone will automatically boot from microSD if a bootable card is inserted. Although it is technically possible to use any ARM distribution (because the PinePhone uses the mainline kernel), there are a few that are designed specifically for mobile use on devices like the PinePhone.

Software releases

The PinePhone Software Releases page has a complete list of currently supported phone-optimized Operating System images that work with the PinePhone as well as other related software information. As soon as more patches get mainlined and distributions ship with the updated kernel, they will also be able to run unmodified on the device. To update any installed operating system please see PinePhone Updating Instructions.

Installation instructions

The instructions to install an operating system to the internal memory ("eMMC") or a microSD card can be found here: PinePhone Installation Instructions.

Troubleshooting

If the PinePhone is not booting from eMMC and/or microSD card anymore it can have two causes:

The battery is drained

If the battery is fully drained, most operating systems and distributions won't boot anymore. One of the exceptions which still boots is the utility *Jumpdrive*, which can usually be used to expose the eMMC and microSD card as drives to a USB-connected computer. Mind that JumpDrive won't expose the eMMC and microSD card with a drained battery but it still can be flashed and booted from microSD card to confirm that the phone still functions and boots up fine.

JumpDrive can be downloaded from here (https://github.com/dreemurrs-embedded/Jumpdrive/releases/download/0.8/pine64-pinephone.img.xz) (not compatible with the PinePhone Pro!).

> **Note:** If JumpDrive boots and other releases do not boot, the battery is likely drained. In that case let it charge while running JumpDrive with a compatible charger for multiple hours and make sure to not fully drain the battery in the future anymore, as that significantly reduces the battery lifetime.

Alternatively to testing JumpDrive, the issue can also be diagnosed by checking the battery charge. Simply remove the battery from the device and measure the voltage on the (+) and (-) contacts on the battery. Make sure to not shorten the contacts as shorting battery pins is a severe life and safety danger.

The installation is corrupted or incorrect

If the installation of the prioritized boot medium is corrupted or incorrect, the PinePhone will not boot anymore. The following problems are common:

- The microSD card is in the wrong slot (see first time installation)
- The image file was flashed to partition 1 (example: *sdx1*, *nvme0x1p1*) instead of the whole device (example: *sdx*, *nvme0x1*)

- An image without bootloader was flashed (mind the *Tow-Boot* bootloader requirements of *Mobian* and *postmarketOS*)
- The operating systems got corrupted, for example after running updates (reflash the device, see PinePhone Installation Instructions)
- An incompatible image was flashed (make sure that the images are compatible. Images for the PinePhone Pro won't boot on the PinePhone and vice versa. Check your invoice to see if you ordered a PinePhone or a PinePhone Pro).

Specifications

- **Dimensions:** 160.5 x 76.6 x 9.2mm
- **Weight:** Between 180 ~ 200 grams
- **SIM Card:** Micro-SIM
- **Display:**
  - **Size:** 5.95 inches (151mm) diagonal
  - **Type:** HD IPS capacitive touchscreen, 16M colors
  - **Resolution:** 1440x720, 18:9 ratio
- **System on Chip:** Allwinner A64 (https://linux-sunxi.org/A64)
- **RAM:** 2GB or 3GB LPDDR3 SDRAM
- **Internal Storage:** 16GB or 32GB eMMC, extendable up to 2TB via microSD, supports SDHC and SDXC
- **Back Camera:** Single 5MP, 1/4", LED Flash
- **Front Camera:** Single 2MP, f/2.8, 1/5"
- **Sound:** Loudspeaker, 3.5mm jack & mic (jack doubles as hardware UART if hardware switch 6 is deactivated)
- **Communication:**
  - **Modem:** Quectel EG25-G (https://www.quectel.com/product/lte-eg25-g/)
  - **LTE-FDD**: B1, B2, B3, B4, B5, B7, B8, B12, B13, B18, B19, B20, B25, B26, B28
  - **LTE-TDD**: B38, B39, B40, B41
  - **WCDMA**: B1, B2, B4, B5, B6, B8, B19
  - **GSM**: B2, B3, B5, B8 (850, 900, 1800, 1900 MHz)
  - **WLAN:** Wi-Fi 802.11 b/g/n, single-band, hotspot
  - **Bluetooth:** 4.0, A2DP
  - **GNSS:** GPS/GLONASS/BeiDou/Galileo/QZSS, with A-GPS
- **Sensors:** Accelerometer, gyroscope, proximity, ambient light, compass
- **Privacy switches:** Modem, WiFi & Bluetooth, Microphone, Cameras
- **Battery:** Lithium-ion, rated capacity 2800mAh (10.64Wh), typical capacity 3000mAh (11.40Wh) (nominally replaceable with any Samsung J7 form-factor battery)

- **I/O:** USB Type-C, USB Host, DisplayPort Alternate Mode output, 15W 5V 3A Quick Charge, follows USB PD specification

Components

Component	Model
Touchscreen	Goodix GT917S
Rear camera	OmniVision OV5640
Camera flash	Awinic AW3641EDNR
Front camera	GalaxyCore GC2145
LCD	Xingbangda XBD599
WiFi	Realtek RTL8723CS
Bluetooth	Realtek RTL8723CS
Modem	Quectel EG25-G
GNSS/GPS	Quectel EG25-G
Magnetometer	ST LIS3MDL
Ambient light / Proximity	SensorTek STK3335
Accelerometer / Gyroscope	InvenSense MPU-6050
Vibration motor	Unknown model
Notification LED	LED0603RGB
Volume buttons	Buttons connected to the KEYADC
Power button	X-Powers AXP803
Battery fuel gauge	X-Powers AXP803

See the PinePhone Component List.

Hardware revisions

The following are all hardware revisions of the PinePhone that have existed, ordered by the time of their releases:

- Project Anakin - Development kit
- Project Don't be evil - Development kit
- PinePhone v1.0 - Developer Edition
- PinePhone v1.1 - Braveheart Edition
- PinePhone v1.2 - Ubports Community Edition
- PinePhone v1.2a - postmarketOS Community Edition

- PinePhone v1.2b - Manjaro Community Edition, KDE Community Edition, Mobian Community Edition and Beta Edition

Hardware accessory

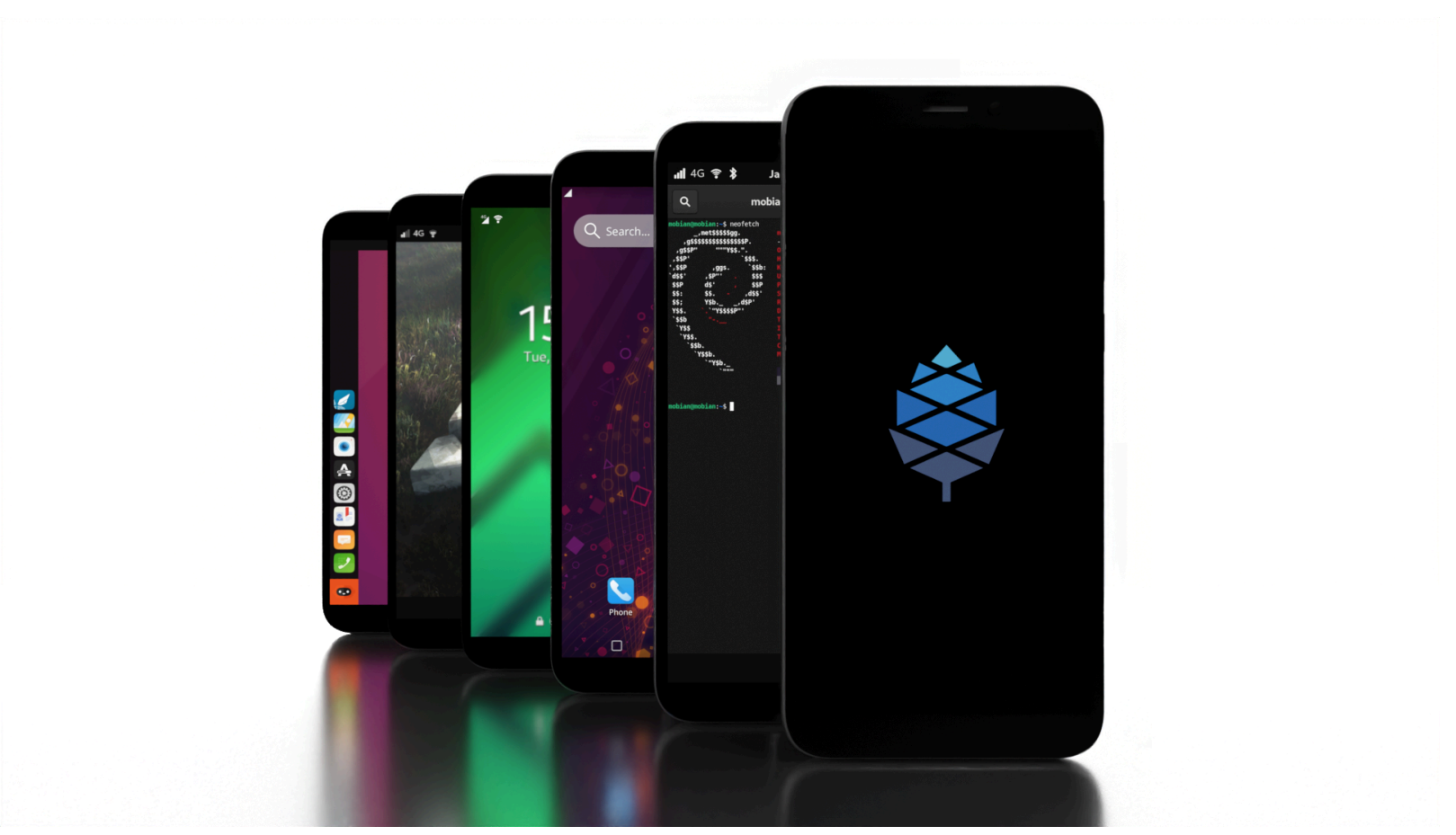

The history of the PinePhone CEs

Add-ons

The PinePhone (and PinePhone Pro) is compatible with the official add-on cases, such as the keyboard, the LoRa add-on, the Qi wireless charging add-on and the fingerprint reader add-on. Details can be found under:

- PinePhone (Pro) Add-ons
- PinePhone (Pro) Keyboard

PinePhone Accessories

See PinePhone Hardware Accessory Compatibility for a list of devices working with the PinePhone (depending on their OS support).

The USB-C can be used to power the device, and offers USB2 host and OTG capabilities, and also can make use of the USB-C capability to integrate HDMI signals. Some USB-C hubs are available that offer power throughput, USB connection, an HDMI port and Ethernet connection.

Pogo pins

The PinePhone has six pogo pins on the back allowing for custom hardware extensions such as wireless charging, an IR blaster, a keyboard extension or extended battery case. The pogo pins provide access to an interrupt line, power inputs/outputs and an I2C interface.

Interrupt	SDA	SCL
DCIN	USB-5V	GND

The DCIN pogo pin is connected to the VBUS line of the phone's USB Type-C connector, serving as another 5 V power input to the phone. However, the PinePhone may act as a USB host (providing 5 V at the USB Type-C connector's VBUS line to a connected device) or as a USB device (drawing from a 5 V source on the USB Type-C connector's VBUS line). Thus, connecting a 5 V power supply to DCIN in order to power the phone may not be safe to do in all conditions, e.g., when the phone is acting as a USB host to a connected USB device.

The USB-5V pogo pin is to used as an "always on" power output from the PinePhone. Depending on a number of factors, USB-5V may be at the battery voltage (between 3.0 V and 4.3 V), or at 5 V. Thus, if you are using USB-5V to power your pogo-pins expansion board, you will probably need to use DC/DC converters/regulators as appropriate. USB-5V is on even while the A64 SoC is powered down.

Pine Store offers the PinePhone Flex Breakout Board (https://pine64.com/product/pinephone-flex-break-out-board/?v=0446c16e2e66). With its 2.54 mm pitch, this breakout may have leads soldered directly to the contacts for use in a solderless breadboard. A non-soldered solution would be to use a TE AMP Connector (https://www.digikey.com/en/products/detail/te-connectivity-amp-connectors/5-520315-6/2258879) that accepts a flat flexible cable with 2.54 mm pitch.

The following community projects use the pogo pin connections:

- The *PinephonePogoBreakout* (https://github.com/SMR404/PinephonePogoBreakout) breakout board from the community member *SMR404*
- Martijn Braams' *Making a backcover extension for the PinePhone* (https://blog.brixit.nl/making-a-backcover-extension-for-the-pinephone/)

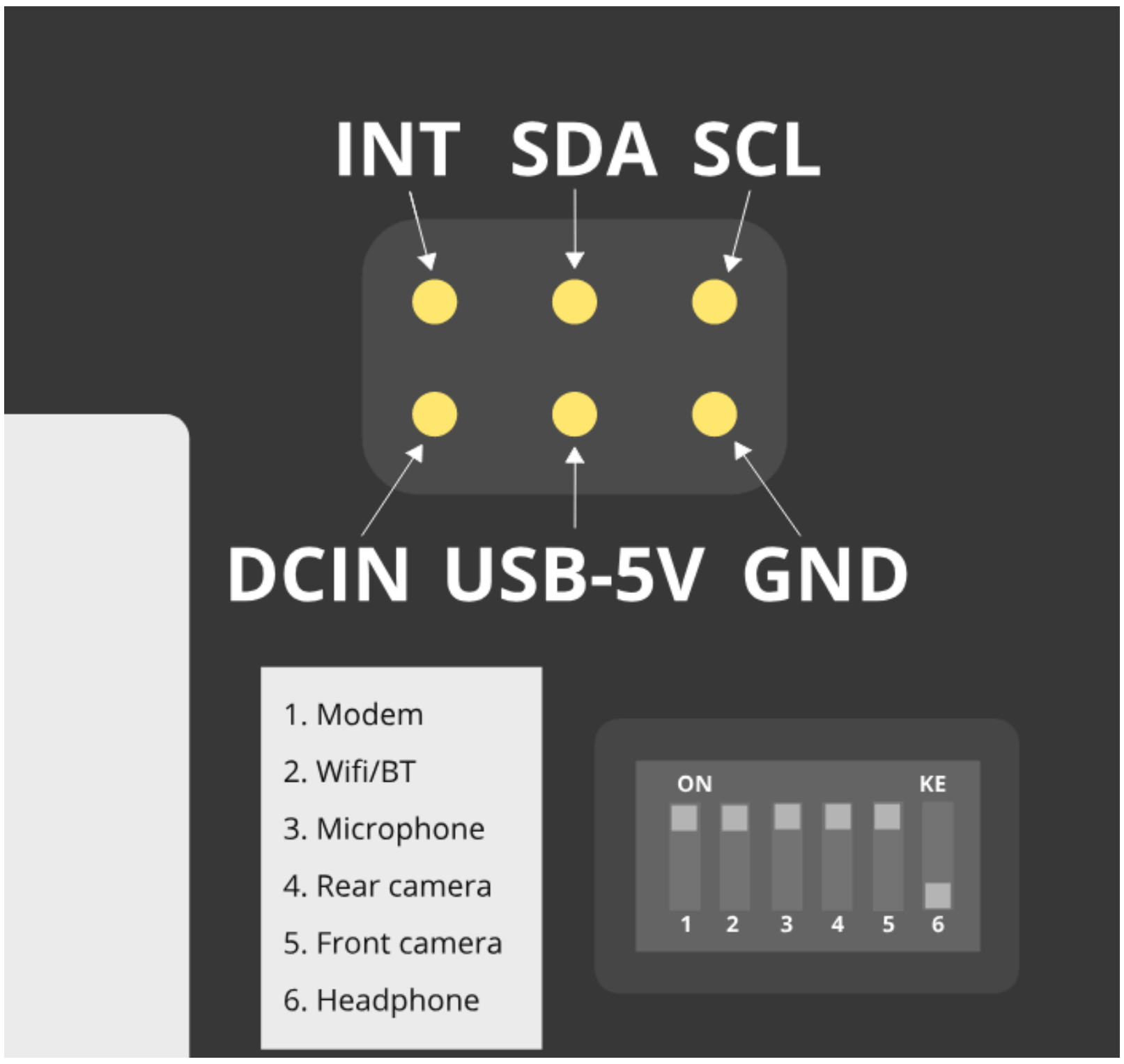

The pogo pins, as visible under the back cover.

Back cover

A step file for the back cover for creating custom cases is freely available here (https://files.pine64.org/doc/PinePhone/PinePhone%20Back%20Cover%20ver%200.5.stp).

Serial console

The PinePhone has a serial port in the headphone connector, it's activated by the 6th contact on the dipswitch. If the switch is set to "on", the headphone connector is in audio mode, if it is set to "off" it's in UART mode. The UART serial connection can also be used for communication with other devices from the PinePhone.

The UART is 115200n8.

The pinout for the serial connector is:

- Tip: RX

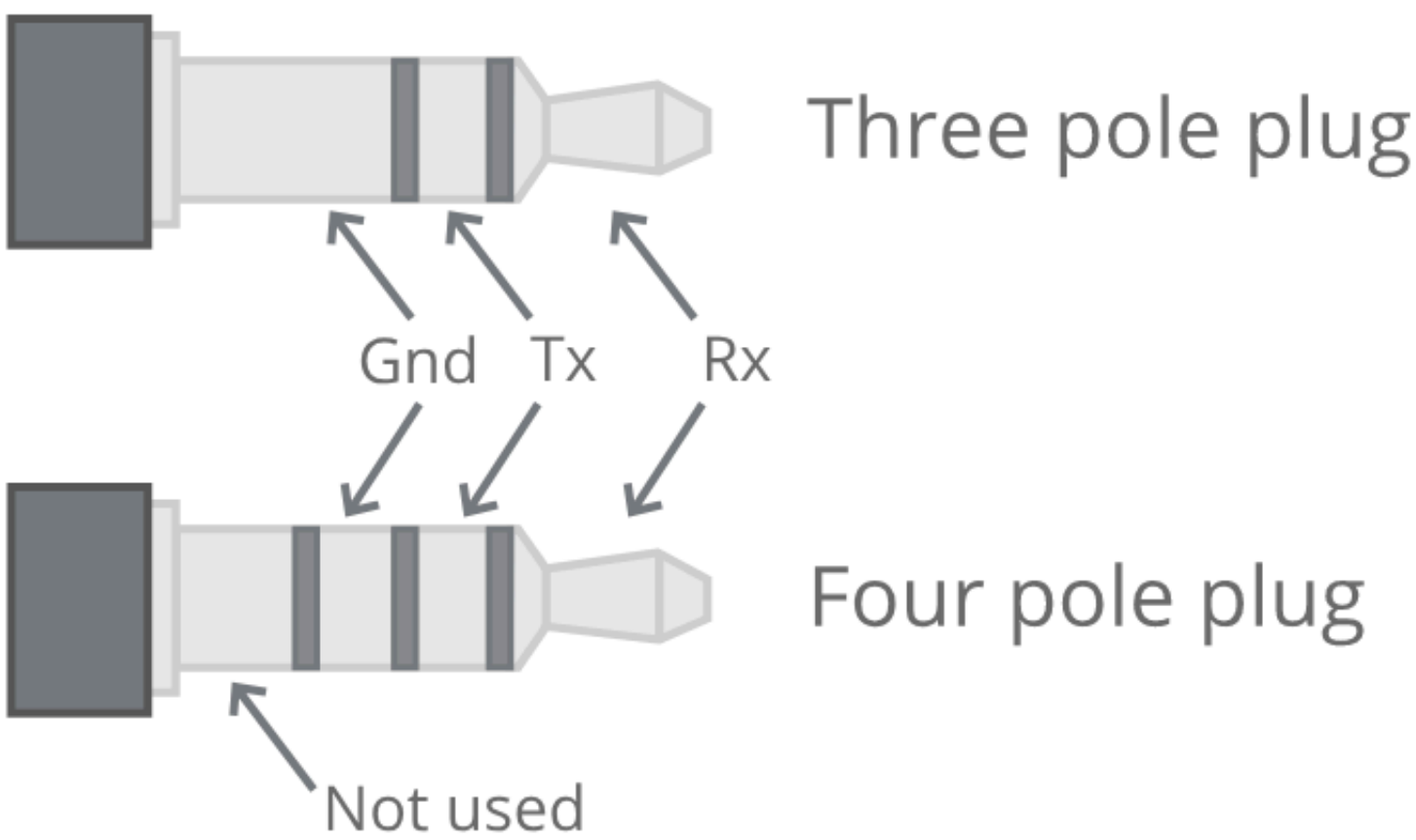

Pinout of the serial adapter. This shows signals from the PinePhone's point of view, so connect the adapter's Rx to Ring 1 and Tx to Tip

- Ring: TX
- Sleeve: GND

You can buy a serial debug cable from the PINE64 Store (https://pine64.com/product/pinebook-pinephone-pinetab-serial-console/). The store cable uses a 4 ring plug, as seen in the here (https://files.pine64.org/doc/pinebook/guide/Pinebook_Earphone_Serial_Console_Developer_Guide.pdf), but a 3 ring plug works just as well. The cable uses a CH340 chipset based serial to USB converter, but any 3.3v serial connection can be used. Because it is a "host"/DTE it means that you need a *cross modem cable* (Null Modem (https://en.wikipedia.org/wiki/Null_modem)) with TX on Tip to be connected to RX. A cable like e.g. FTDI TTL-232R-3V3-AJ (https://www.ftdichip.com/Products/Cables/USBTTLSerial.htm) which has TX on Tip and RX on Ring fits perfectly.

Privacy switch configuration

The PinePhone features six switches that can be used to configure its hardware. They are numbered 1-6, with switch 1 located nearest to the modem. Their "on" position is toward the top of the phone.

Picture of the privacy switches

Number	Name	Explanation	Description
1	Modem	Pulls Q1501 gate up (FET killing modem power)	"On" enables 2G/3G/4G communication and GNSS hardware, "off" disables it.
2	WiFi / Bluetooth	Pulls up CHIP_EN	"On" enables WiFi and Bluetooth communication hardware, "off" disables it.
3	Microphone	Breaks microphone bias voltage from the SoC	"On" enables audio input from on-board microphones (not 3.5 mm jack), "off" disables it.
4	Rear camera	Pulls up PWDN on OV5640	"On" enables the rear camera, "off" disables it.
5	Front camera	Pulls up PWDN on GC2145	"On" enables the front camera, "off" disables it.
6	Headphone	Pulls up IN2 on analog switch BCT4717ETB	"On" enables audio input and output via the 3.5 mm audio jack, "off" switches the jack to hardware UART mode.

Camera

The PinePhone has two cameras, OmniVision OV5640 with 5MP (up to 2592 x 1944 pixels) as rear camera and GalaxyCore GC2145 with 2MP (up to 1600 x 1200 pixels) as front camera.

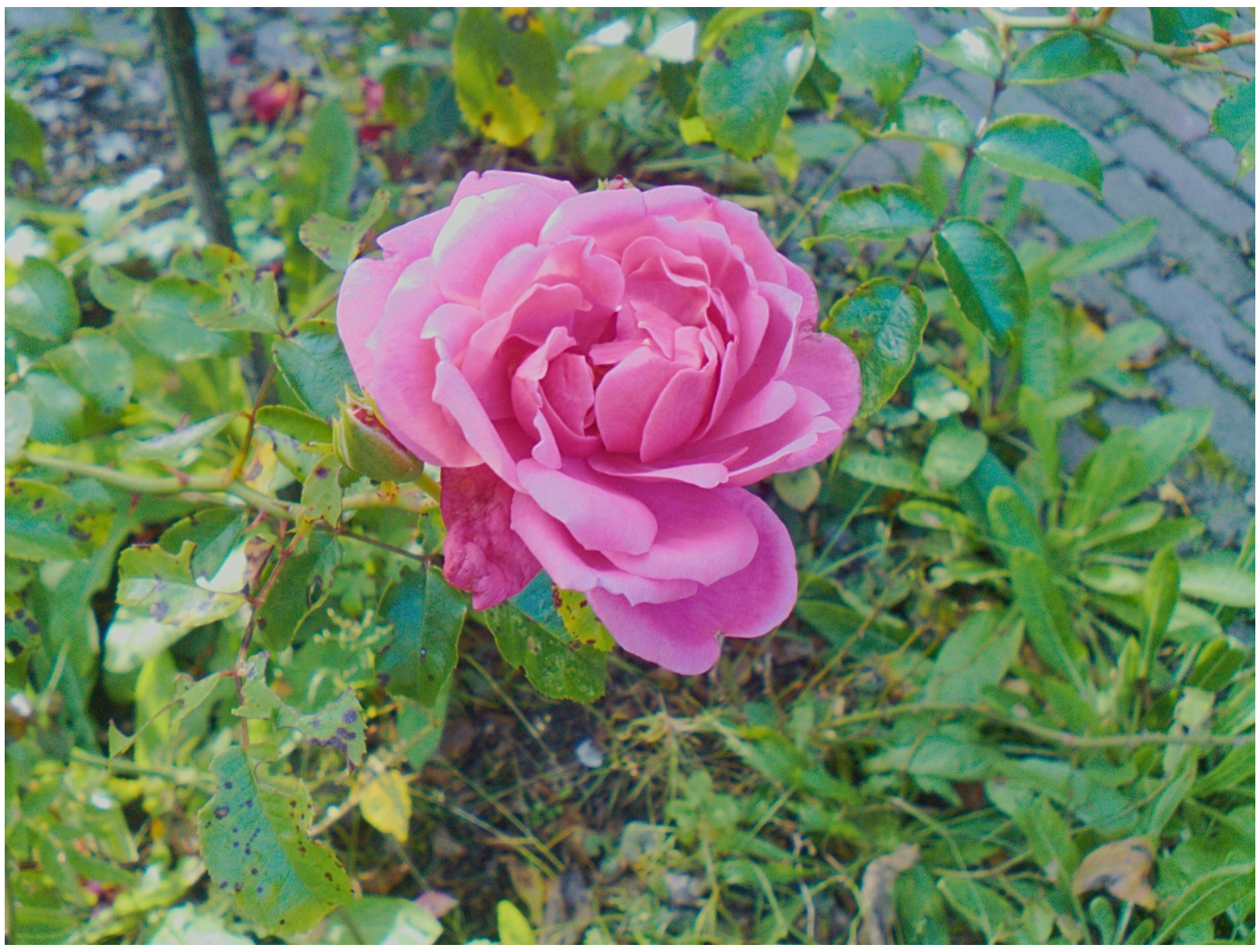

Example picture taken on the PinePhone's rear camera by Martijn Braam using his app *Megapixels*.

Further details regarding the camera and the Megapixels camera app can be found on Martijn's blog (https://blog.brixit.nl/tag/phones/).

Battery

The phone ships with a protective plastic sticker between the battery and the phone to protect the device from turning on during shipping. You need to gently open the back cover, then remove the battery and finally remove the sticker and check that the pins aren't bent. Note: If the battery is stuck inside the phone, the mid screw in the lower part of the midframe needs to be slightly loosened, see here.

> **Note:** The EG25-G modem and the RTL8723CS WiFi and Bluetooth do not work without a battery and with a drained battery, even when enough power is supplied to the PinePhone via the USB Type-C port. Most operating systems won't boot without a battery or with a drained battery.

The supplied battery (https://files.pine64.org/doc/datasheet/pinephone/PinePhone%20QZ01%20Battery%20Specification.pdf) is meant to be compatible with Samsung part number EB-BJ700BBC / BBE / CBE from the 2015 J7 phone. The extended life aftermarket BBU does fit, although it is a tight fit.

Warning: Using an aftermarket battery with a higher capacity is done at own risk. Batteries with a higher capacity especially in combination with an external charger can lead to overvoltage, which fries the modem and/or the Bluetooth and WiFi chip.

The battery terminals, from the nearest to the battery edge to the nearest to the middle of battery, are as follows:

+ve	thermistor	-ve	not connected

The battery includes a protection circuit that isolates it in a number of fault conditions, including if it is discharged too far. The fully discharged battery can be recharged by connecting the phone to a charger with a sufficient output. Once it has charged sufficiently you will be able to boot the phone.

Modem

The PinePhone uses Quectel EG25-G as modem. AT commands are used to communicate with the modem.

AT commands

A list of documented AT commands can be found for example in this AT commands documentation from Quectel. Further undocumented AT commands found by the developer megi, who reverse-engineered parts of the modem and its firmware, can be found on megi's website here (http://xnux.eu/devices/feature/modem-pp-reveng.html#toc-un-der-documented-at-commands).

To send AT commands to the modem under Linux, `minicom` or the often-preinstalled `atinout` utility can be used.

atinout example (use single quotes instead of double quotes around the command if the command contains double quotes):

```
echo "AT+<command here>" | sudo atinout - /dev/ttyUSB2 -
```

minicom example (you may need to use sudo too):

`minicom -D /dev/ttyUSB2`

VoLTE

The PinePhone's modem supports VoLTE and comes with a few VoLTE profiles preloaded. Most operating systems try to set the correct profile automatically.

To list the available VoLTE profiles:

```
AT+QMBNCFG="list"

+QMBNCFG: "List",0,0,0,"ROW_Generic_3GPP",0x0501081F,202112292
+QMBNCFG: "List",1,0,0,"VoLTE-ATT",0x0501033C,202105171
+QMBNCFG: "List",2,0,0,"hVoLTE-Verizon",0x05010141,202111151
+QMBNCFG: "List",3,0,0,"Sprint-VoLTE",0x05010205,202010201
+QMBNCFG: "List",8,0,0,"Reliance_OpnMkt",0x05011B38,202003251
+QMBNCFG: "List",9,0,0,"TF_Germany_VoLTE",0x05010C1B,202004151
+QMBNCFG: "List",10,0,0,"TF_Spain_VoLTE",0x05010CFA,202106171
+QMBNCFG: "List",11,0,0,"Volte_OpenMkt-Commercial-CMCC",0x05012071,202201063
+QMBNCFG: "List",12,0,0,"VoLTE_OPNMKT_CT",0x050113FC,202201063
+QMBNCFG: "List",13,0,0,"CU-VoLTE",0x05011508,202201063
+QMBNCFG: "List",14,1,1,"Telstra-Commercial_VoLTE",0x0580079E,202112221
+QMBNCFG: "List",15,0,0,"Commercial-KDDI",0x0501071D,202011192
+QMBNCFG: "List",16,0,0,"Commercial-DCM",0x05010D17,202111091
+QMBNCFG: "List",17,0,0,"Commercial-SKT",0x05012715,202103121
+QMBNCFG: "List",18,0,0,"Commercial-KT",0x05012C0D,202108241
+QMBNCFG: "List",19,0,0,"Commercial-LGU",0x05012626,202108241
+QMBNCFG: "List",20,0,0,"Commercial-USCC",0x0504FC40,202011111
+QMBNCFG: "List",21,0,0,"Optus-Commercial_VoLTE",0x05800CA2,201910241
+QMBNCFG: "List",22,0,0,"STC_Saudi_VoLTE",0x0501FE01,201912231
+QMBNCFG: "List",23,0,0,"Commercial-Rogers",0x05018821,202108021
```

The above are the profiles for firmware version EG25GGBR07A08M2G_30.006.30.006 and may not reflect the profiles on other firmware versions. If a profile is required that is shown

above, but not in AT+QMBNCFG="list", then you may need to update your modem's firmware to a newer version as detailed below.

To select a profile manually, select the best fitting one or a generic one if none fits:

```
AT+QMBNCFG="select","ROW_Generic_3GPP"
```

Then enable Voice over LTE using:

```
AT+QCFG="ims",1
```

And reboot the modem to apply the settings:

```
AT+CFUN=1,1
```

To check the status of VoLTE during a call, the AT command `CLCC` can be used:

```
AT+CLCC

+CLCC: 1,1,0,1,0,"",128
+CLCC: 2,1,0,1,0,"",128
```

In the fourth item of the list, "0" means voice and and "1" means data. If both rows have "1" then the voice call is being carried over VoLTE.

APN settings

The APN setting is only required for a public Internet connection ("data") on the phone. For tested APN settings and how to apply them see PinePhone APN Settings.

Carrier support

The page PinePhone Carrier Support contains information about the frequency support of different carriers and hints on setting up cellular network connectivity.

Documents

Detailed information about the modem can be found on the page of the developer megi (https://xnux.eu/devices/feature/modem-pp.html#toc-modem-on-pinephone), including reverse-engineered parts of the firmware and its functions. There is also a document about using the modem from January 18th 2020 by megi here (https://megous.com/dl/tmp/modem.txt). A script at the end of the document showcases a way to poweroff the modem before powering off the phone, which is integrated into most of the available operating systems.

Firmware update

There is a (nearly) free custom firmware and the stock firmware available for the PinePhone. Both can be updated to a newer version with new features and bug fixes.

Custom firmware

There is a (nearly) free custom firmware for the PinePhone modem by the developer *biktorgj*, which can be found here (https://github.com/the-modem-distro/pinephone_modem_sdk).

The custom firmware has various advantages (and zero disadvantages) over the stock firmware, including:

- Signal tracking support with checks against the OpenCelliD database
- Persistent storage is optional and unexpected shutdowns don't mess up the modem
- A lower energy consumption due to the lower minimum clock frequency
- And many more, see Features not available on stock firmware (https://github.com/the-modem-distro/pinephone_modem_sdk#features-not-available-on-stock-firmware)

The custom firmware can be flashed using fwupd (https://wiki.postmarketos.org/wiki/Fwupd#Upgrading_Modem_Firmware_on_the_PinePhone_.28Pro.29) or a flashing script (https://github.com/the-modem-distro/pinephone_modem_sdk/blob/kirkstone/docs/FLASHING.md).

Stock firmware

> The following instructions are directed towards professional users. It is highly recommend to make sure the update process is not interrupted to prevent the modem from bricking.

The stock modem firmware can be updated to a newer version if it is outdated. The firmware version can be checked using the following AT command (at the example of `atinout`, alternatively `minicom` can be used to communicate with the modem too):

```
echo 'AT+QGMR' | sudo atinout - /dev/ttyUSB2 -
```

Pre-update checklist:

Please make sure all requirements of the checklist are fulfilled. If the update process is interrupted it will lead to a corrupted firmware of the modem, causing it to brick. Recovering a bricked modem is exponentially more complicated and requires the user to boot a special mode by physically bridging test points on the modem.

- The battery needs to be charged sufficiently
- The phone needs to be plugged into a charger
- Deep sleep is recommended to be disabled as it can interrupt the update process
- It is recommended to close all other running applications
- Use common sense while doing the update, don't do the update while being impaired in any way

To get the latest firmware, clone the repository of user Biktorgj on the phone:

```
git clone https://github.com/Biktorgj/quectel_eg25_recovery
```

After cloning the directory, open it with cd:

```
cd quectel_eg25_recovery
```

Then run qfirehose, which starts the flashing process:

```
sudo ./qfirehose -f ./
```

The modem will automatically reboot after the update process is done. The boot process takes around 30 to 60 seconds. After that it is highly recommended to reboot the device.

Firmware modifications

See PineModems for more information regarding modem bootloader unlocking, building a custom modem firmware and modem recovery.

GPS / GNSS

The GPS engine in the modem supports mutli-GNSS reception from GPS, GLONASS, BeiDou, Galileo and QZSS independent of a cellular connection. The operation of the GNSS subsystem can be controlled via a separate set of AT commands, or via qmi. The A-GPS data upload uses the file management AT commands, which also have their own manual. These are linked in the documentation section below.

As with most smartphones, the PinePhone has a small antenna and has difficulty getting a first fix without assistance data, a cold start can take 15 minutes under good conditions. The *eg25-mananger* is configured to upload A-GPS data by default (see here (https://gitlab.com/mobian1/eg25-manager/-/merge_requests/15)).

Basic testing of GNSS reception can be done by using the AT command interface (*/dev/ttyUSB2*) from a terminal program like *minicom* and the data output interface (*/dev/ttyUSB1*) to feed NMEA data into gpsmon or some other program that can parse standard NMEA sentences.

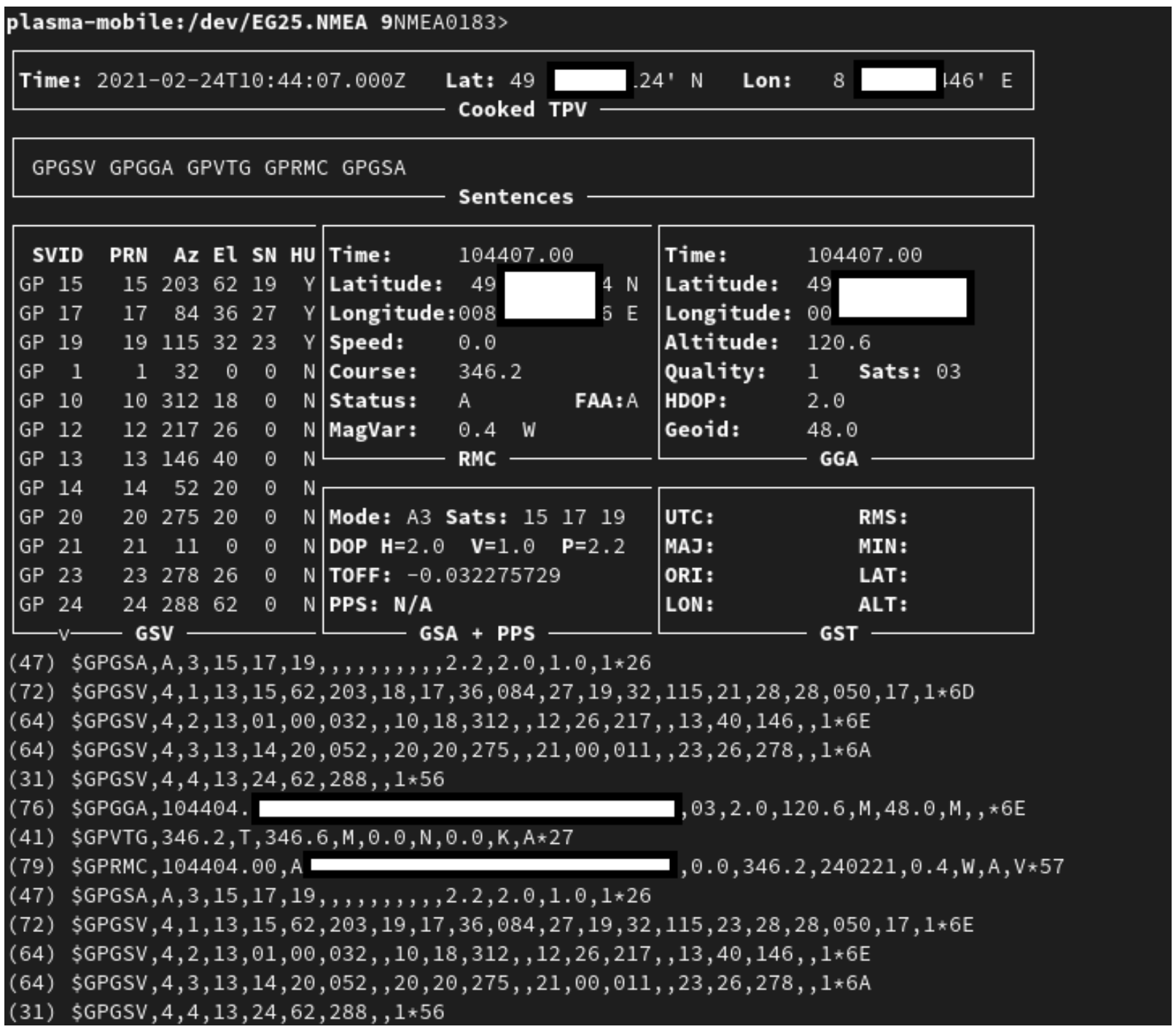

gpsmon decoding GPS data from */dev/ttyUSB1*

To check if GNSS data output is enabled, you can

```
cat /dev/ttyUSB1
```

this should display a stream of NMEA sentences

```
$GPVTG,,T,,M,,N,,K,N*2C
$GPGSA,A,1,,,,,,,,,,,,,,,,*32
$GPGGA,,,,,,0,,,,,,,,*66
```

Further details can be found under PinePhone Sensors and Navigation.

Voice mail

The operating systems of the PinePhone may not have support for accessing your voicemail by holding down the 1-key. Carriers might support accessing the voice mail via an external number however.

Safety

General recommendations

Due to the greater control the user is having over the device and its software comes also greater responsibility. It is necessary to verify the configuration of the device to make sure that responsible settings are used. The different operating systems may come with non-sane default settings, including SSH with weak password authentication being enabled by default and exposed to the public Internet, the absence of a firewall, default passwords, unencrypted files, too high temperature zones and emergency shutoff values or an enabled root account. The usage of public resources to verify such settings (such as the in case of GNU/Linux, the Arch Linux security (http

s://wiki.archlinux.org/title/security) wiki page, or the general recommendations (https://wiki.archlinux.org/title/general_recommendations)) as well as the corresponding operating system's or distribution's resources are strongly recommended.

Charging safety

The PinePhone supports up to 5V 3A (15W) Quick Charge, it follows the USB Power Delivery specification. Only compatible phone chargers may be used, charging the phone with incompatible chargers (for example laptop chargers with a higher voltage) is prohibited. Charging the phone releases heat, general safety recommendations must be followed, see the section *Thermal safety*.

Thermal safety

With the Allwinner A64 being an older generation SoC with a large 40nm chip, the phone produces quite some heat with medium or higher use and especially also during charging or when using USB accessories, like a docking station. Measurements to prevent damage to the phone and to its surroundings need to be taken by the user. This includes especially a proper handling of the phone: do not charge the phone in a way where heat builds up around the phone without being able to escape. Especially don't charge your phone under a pillow, blankets, in pockets or bags. Charging the phone produces heat and charging the phone in a way, where the excessive heat can't dispose around the phone poses an immediate fire risk.

The user might notice that the phone gets warm under usage, compared to phones with more up-to-date hardware. Under normal circumstances these temperatures don't pose a risk while being in the levels within the safe operating temperatures (which lay far beyond the point where components can be too hot to touch). Higher temperatures might especially be experienced on the top side of the screen and on the inside of the phone at the RF shield of the modem. The higher temperature of the RF shield of the modem is commonly caused by the SoC on the opposite side of the mainboard, the RF shield of the modem is used to disperse heat of the SoC. In newer mainboard revisions starting from 1.2a there are also thermal pads on the back cover and between the SoC's RF shield and the screen, dispersing heat on the screen and on the back cover. In the past there has been safety issues regarding thermal safety functions, causing temperature reads to not properly work over an extended period of time, which was causing heat damage in some cases (see the documentation of that issue by the developer Megous here (http://xnux.eu/log/#018) and here (http://xnux.eu/log/#017)). While the developers are working hard to prevent such issues, they can't be excluded under all circumstances (see state of the software). The users are expected to monitor their phones' thermal safety at every point at this state of the software.

It is highly recommend to update the phone on a regular basis to always get the latest improvements. The default settings to throttle the performance and to shut down the phone when reaching critical temperatures might be set to a too high point depending on the specific usage and usage length. Under GNU/Linux the phone's thermal management behavior can be modified via the Thermal Sysfs driver to achieve lower temperatures and preventing the screen and other components to potentially take damage, see PinePhone Thermal Tweaks for the details.

Frequently asked questions

For a list of frequently asked questions (including information regarding the shipping) see PinePhone FAQ.

Modifications and repairs

Swapping in a new mainboard revision

The mainboard can be replaced, for example for upgrading to a newer hardware revision or if it is faulty. The replacement board does not have an OS preinstalled, to test if everything is working after swapping the mainboard a flashed SD card is required. The mainboard also comes with a non-functional firmware on the ANX chip, a newer firmware version has to be flashed as explained below to get certain USB functionality to work.

Replacing the mainboard

> Replacement boards come with an empty eMMC, which means that trying to boot from them looks like the board is faulty (no LEDs, no screen, no reaction of the phone). Please boot an OS from SD card.

Prior to replacing your PinePhone's mainboard please read the steps outlined in bullet points below and watch the attached video.

1. You' ll need a small Phillips screwdriver and a prying tool to swap out the PinePhone' s mainboard.
2. Remove the PinePhone' s back cover. See your quick start guide for details.
3. Remove the battery as well as any inserted SD and SIM cards.
4. Unscrew all 15 Phillips head screws around the midframe of the phone.
5. Gently pry up the midframe using a guitar pick or credit card corner. It is easiest to separate the midframe at one of the bottom edges. Work your way around all the sides of the phone until the midframe separates from the phone' s body.
6. Detach all ribbon cables and "Lego" connectors. List of things to detach: 1) two "Lego" connects at the bottom of the mainboard. 2) u.FL antenna connect and touchscreen digitizer on PCD left side. 3) LCD ribbon cable top of mainboard, next to audio/ UART jack.
7. Pry the mainboard up gently from the left-hand side.
8. Remove front and main cameras and reset them into the new mainboard.
9. Check that the rubber proximity sensor housing is in the chassis, not stuck to the removed mainboard.
10. Place the new mainboard in the chassis, hooking in on the plastic tabs on left side and pressing down firmly on opposite side, and follow the steps (7-2) in reverse. When reattaching the midframe take care that no cables are out of place or trapped, as they may be damaged when tightening screws.

After swapping the mainboard the phone won't boot as there is no OS on the replacement board's eMMC preinstalled. To boot an OS insert a flashed SD card.

A video tutorial by *Martijn Braam* can be found here (or alternatively a video tutorial by user *brigadan* with additional notes about the camera swap and proximity sensor isolator here (https://www.youtube.com/watch?v=J3AJEF7akkw)):

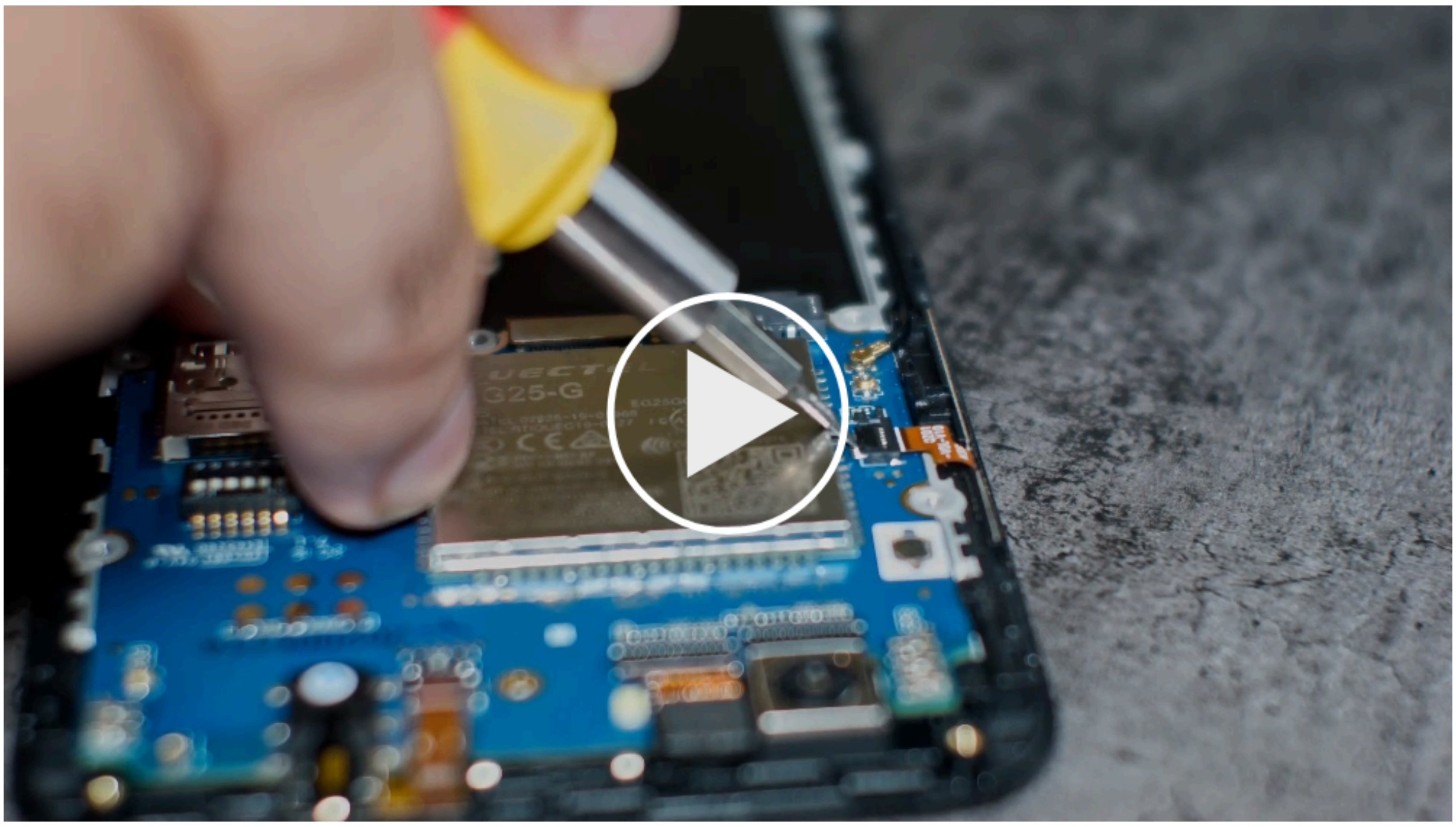

Watch Martijn Braam's video tutorial here: https://www.youtube.com/watch?v=5GbMoZ_zuZs.

Flashing the ANX firmware

Method 1

After swapping the mainboard the ANX7688 chip has to be flashed for full USB functionality.

Under GNU/Linux this can be done by downloading the latest ANX7688 firmware image on the phone:

```
wget https://xff.cz/git/linux-firmware/plain/anx7688-fw.bin
```

and executing as root ("sudo su") on the phone:

```
cp anx7688-fw.bin /lib/firmware/
echo 1 > /sys/class/typec/port0/device/flash_eeprom
```

Method 2

Booting a factory test image will automatically flash the ANX7688 chip. See Factory Test OS for such an image.

Replacing the screen

Before attempting to replace the screen be sure to review the section on replacing the mainboard since that will get you most of the way there. Be aware that the replacement screen is actually the entire front frame of the phone and there are components that will need to be swapped from your old screen.

- Make sure you have a precision screwdriver set that has the correct size Philips tip. The screws are very small and the heads can easily be stripped if the screwdriver is not correct - if you feel your screwdriver slipping, stop what you are doing and try one that is a better fit. A magnetized screwdriver will help in not losing screws, as will a magnetic parts holder to keep them in while working.

- There are a number of components and cables as well as the insulator sheet under the battery that are glued in place. A hair dryer will loosen the glue and make them much easier to remove. You may want to order extra cables along with the screen just in case.

- The vibration motor, which is part of the USB-C board assembly and glued into place, will come apart easily and be damaged if you pry it up in the wrong place. Make sure you pry from underneath the complete part, not midway on its housing. The ribbon cable attaching this to the USB-C board is small, thin, and fragile so be careful with that as well.

- The new screen comes with new side switches and insulator sheet but there are a number of parts that need to be transferred from the old screen, like the thin coax cable running up the side, the phone ear speaker, proximity sensor gasket, and a gold-colored mesh glued in place that needs to be transferred to a flexible circuit included on the new screen. If you don't swap over the proximity sensor rubber gasket the screen will immediately turn off after logging in. Be careful when routing the coax cable that it goes around the screw holes or you may drive a screw right through the cable.

Take your time, use the right tools, be careful and you should be rewarded with success.

Spare parts not available in the PINE64 store

- Earpiece: See PinePhone (Pro) replacement earpieces
- Loudspeaker dimensions: 15x11x3 mm. Compatible with Nokia N91, Lenovo A536 (requires soldering) and others, see here (https://forum.pine64.org/showthread.php?tid=12046&pid=85698#pid85698)
- Proximity sensor rubber isolator

Other hardware issues

See also PinePhone Hardware Issues for more issues and how-to's on hardware components including the sim reader, the internal microphone, and the bottom speaker.

Press

For an overview about media of the PinePhone you can use for the news, blogs, or similar see PinePhone Press.

PinePhone board information, schematics and certifications

PinePhone case 3D STP file:

- PinePhone and PinePhone Pro case 3D case, please note that this release as-is (https://files.pine64.org/doc/PinePhone/PinePhone_and_Pro_case_asm.stp)

PinePhone mainboard schematic:

- PinePhone mainboard Released Schematic ver 1.2b (https://files.pine64.org/doc/PinePhone/PinePhone%20v1.2b%20Released%20Schematic.pdf)
- PinePhone mainboard Released Schematic ver 1.2a (https://files.pine64.org/doc/PinePhone/PinePhone%20v1.2a%20Released%20Schematic.pdf)
- PinePhone mainboard Released Schematic ver 1.2 (https://files.pine64.org/doc/PinePhone/PinePhone%20v1.2%20Released%20Schematic.pdf)
- PinePhone schematic ver 1.2 change list
- "Braveheart" PinePhone mainboard Schematic ver 1.1 (https://files.pine64.org/doc/PinePhone/PinePhone%20Schematic%20v1.1%2020191031.pdf)
- "Braveheart" PinePhone mainboard component top placement drawing ver 1.1 (https://files.pine64.org/doc/PinePhone/PinePhone%20mainboard%20top%20placement%20v1.1%2020191031.pdf)
- "Braveheart" PinePhone mainboard component bottom placement drawing ver 1.1 (https://files.pine64.org/doc/PinePhone/PinePhone%20mainboard%20bottom%20placement%20v1.1%2020191031.pdf)
- PinePhone component list

PinePhone USB-C small board schematic:

- PinePhone USB-C small board Schematic ver 1.0 (https://files.pine64.org/doc/PinePhone/PinePhone%20USB-C%20small%20board%20schematic%20v1.0%2020190730.pdf)
- PinePhone USB-C small board component top placement drawing ver 1.0 (https://files.pine64.org/doc/PinePhone/PinePhone%20USB-C%20small%20board%20top%20placement%20v1.0%2020190730.pdf)
- PinePhone USB-C small board component bottom placement drawing ver 1.0 (https://files.pine64.org/doc/PinePhone/PinePhone%20USB-C%20small%20board%20bottom%20placement%20v1.0%2020190730.pdf)

PinePhone certifications:

- PinePhone FCC Certificate (https://files.pine64.org/doc/cert/PinePhone%20FCC%20SDOC%20Certificate-S19112602605001.pdf)
- RF Exposure SAR Information from FCC ID site (https://fcc.report/FCC-ID/2AWAG-PINEPHONE)
- PinePhone CE RED Certificate (https://files.pine64.org/doc/cert/PinePhone%20CE%20RED%20Certificate-S19112602602.pdf)
- PinePhone ROHS Report (https://files.pine64.org/doc/cert/PinePhone%20ROHS%20Report.pdf)
- Note: PinePhone's Type Allocation Code (TAC) is *86769804*

Datasheets for components and peripherals

Allwinner A64 SoC information:

- Allwinner A64 SoC brief introduction (https://files.pine64.org/doc/datasheet/pine64/A64%20brief%20v1.0%2020150323.pdf)
- Allwinner A64 SoC Data Sheet V1.1 (Official Released Version) (https://files.pine64.org/doc/datasheet/pine64/A64_Datasheet_V1.1.pdf)
- Allwinner A64 SoC User Manual V1.0 (Official Release Version) (https://files.pine64.org/doc/datasheet/pine64/Allwinner_A64_User_Manual_V1.0.pdf)

X-Powers AXP803 PMIC (Power Management IC) information:

- AXP803 PMIC datasheet (https://files.pine64.org/doc/datasheet/pine64/AXP803_Datasheet_V1.0.pdf)

LPDDR3 (178 Balls) SDRAM:

- Artmem LPDDR3 datasheet (https://files.pine64.org/doc/datasheet/pinephone/ATL3A1632H12A_mobile_lpddr3_11x11.5_v1.0_1600.pdf)

eMMC information:

- Kimtigo eMMC datasheet (https://files.pine64.org/doc/datasheet/pinephone/Kimtigo_fbga153_16_32_64_eMMC_datasheet_v1.3.pdf)

CMOS camera module information:

- PinePhone 5M Pixel Real CMOS Image Sensor Module (https://files.pine64.org/doc/datasheet/pinephone/QZ01-rear-2019-0717(HW)%20Model.pdf)
- OV5640 5MP CMOS Image Sensor SoC for Rear Module datasheet (https://files.pine64.org/doc/datasheet/pinephone/OV5640_datasheet.pdf)
- OV5640 Embedded Firmware User Guide – VCM AF Module (https://usermanual.wiki/Document/OV5640FirmwareUserGuideV10.952852672.pdf)

- PinePhone 2M Pixel Front CMOS Image Sensor Module (https://files.pine64.org/doc/datasheet/pinephone/QZ01-front-2019-0717(HW)%20Model.pdf)
- GC2145 2MP CMOS Image Sensor SoC for Front Module datasheet (https://files.pine64.org/doc/datasheet/pinephone/GC2145%20CSP%20DataSheet%20release%20V1.0_20131201.pdf)

LCD touch screen panel information:

- 5.99" 1440x720 LCD IPS Panel specification (https://files.pine64.org/doc/datasheet/pinephone/PinePhone%20LCD-QZ01.pdf)
- ST7703 LCD Controller datasheet (https://files.pine64.org/doc/datasheet/pinephone/ST7703_DS_v01_20160128.pdf)
- GOODiX GT917S Capacitive Touch Controller datasheet (https://files.pine64.org/doc/datasheet/pinephone/GT917S-Datasheet.pdf)

Lithium battery information:

- PinePhone Lithium Battery specification (https://files.pine64.org/doc/datasheet/pinephone/PinePhone%20QZ01%20Battery%20Specification.pdf)
- PinePhone Lithium Battery ZCV curve chart (https://files.pine64.org/doc/datasheet/pinephone/PinePhone%20QZ01%20Battery%20ZCV%20Curve%20Chart.xlsx)
- PinePhone Lithium Battery 3D onshape drawing (https://cad.onshape.com/documents/5194d6303f021e6f300b70a0/w/7b63ac4c32ed63dfd78c7840/e/5d43c0ce5b665bae10082a08)

WiFi/BT module information:

- RTL8723BS/RTL8723CS specification (https://files.pine64.org/doc/datasheet/pine64/RTL8723BS.pdf)

LTE module information:

- File:Quectel EG25-G LTE Standard Specification V1.3.pdf
- File:Quectel EG25-G Hardware Design V1.4.pdf
- File:Quectel EC2x&EG9x&EG2x-G&EM05 Series AT Commands Manual V2.0.pdf
- File:Quectel EC2xEG25-GEG9xEM05 FILE AT Commands Manual V1.0.pdf
- File:Quectel EC2x&EG9x&EG2x-G&EM05 Series GNSS Application Note V1.3.pdf

Sensors:

- ST LIS3MDL 3-axis Magnetometer Datasheet (https://www.st.com/en/mems-and-sensors/lis3mdl.html)
- InvenSense MPU-6050 Six-Axis (Gyro + Accelerometer) MEMS datasheet (https://www.invensense.com/products/motion-tracking/6-axis/mpu-6050/)
- SensorTek STK3335 Ambient Light Sensor and Proximity Sensor (https://www.sensortek.com.tw/en/product/Proximity_Sensor_with_ALS.html)

Digital video to USB-C bridge:

- ANX7688 product brief (https://www.analogix.com/en/system/files/AA-002281-PB-6-ANX7688_Product_Brief.pdf)

Case information:

- PinePhone case exploded diagram (https://files.pine64.org/doc/datasheet/pinephone/PinePhone%20Exploded%20Diagram%20ver%201.0.pdf)
- PinePhone back cover 3D file (https://files.pine64.org/doc/datasheet/pinephone/PinePhone%20Back%20Cover.stp)

Other components:

- See the Component List

Resources

The following articles contain more specific information:

- PinePhone Accessibility
- PinePhone Power Management
- PinePhone Security
- PinePhone UBports OS Design Discussion

The following resources have been made available by Ondřej Jirman, a developer for the Pinephone:

- Getting started with PinePhone Hardware (https://xnux.eu/howtos/pine64-pinephone-getting-started.html)
- State of development progress (https://xnux.eu/devices/pine64-pinephone.html)
- Development log (https://xnux.eu/log/)
- PinePhone Technical News and Update, also applies to other Allwinner devices including PINE A64 SBC (https://xnux.eu/news.html)
- Contributions to the kernel development (https://xnux.eu/contribute.html)

External links

- The PinePhone on the official Pine store (https://pine64.com/product-category/pinephone/)
- PinePhone spare parts on the official Pine store (https://pine64.com/product-category/smartphone-spare-parts/)
- PinePhone accessories on the official Pine store (https://pine64.com/product-category/smartphone-accessories/)

Retrieved from "https://wiki.pine64.org/index.php?title=PinePhone&oldid=23430"

This page was last edited on 21 July 2026, at 00:30.
